# *Origanum majorana* Extracts: A Preliminary Comparative Study on Phytochemical Profiles and Bioactive Properties of Valuable Fraction and By-Product

**DOI:** 10.3390/plants14152264

**Published:** 2025-07-23

**Authors:** Simone Bianchi, Rosaria Acquaviva, Claudia Di Giacomo, Laura Siracusa, Leeyah Issop-Merlen, Roberto Motterlini, Roberta Foresti, Donata Condorelli, Giuseppe Antonio Malfa

**Affiliations:** 1 Department of Drug and Health Sciences, University of Catania, Viale A. Doria 6, 95125 Catania, Italy; simone.bianchi@unict.it (S.B.); racquavi@unict.it (R.A.); donatacondorelli@libero.it (D.C.); g.malfa@unict.it (G.A.M.); 2 Research Centre on Nutraceuticals and Health Products (CERNUT), University of Catania, Viale A. Doria 6, 95125 Catania, Italy; 3 Italian National Research Council ICB-CNR, Institute of Biomolecular Chemistry, Via Paolo Gaifami 18, 95126 Catania, Italy; 4 Institut National de la Santé et de la Recherche Médicale (INSERM), Mondor Institute for Biomedical Research (IMRB), Faculty of Health, University Paris-Est Créteil, F-94010 Créteil, France; leeyah.issop-merlen@inserm.fr (L.I.-M.); roberto.motterlini@inserm.fr (R.M.); roberta.foresti@inserm.fr (R.F.)

**Keywords:** polyphenols, Rosmarinic acid, cancer, sustainability, inflammation, oxidative stress, cytochrome P450 isoforms

## Abstract

*Origanum majorana* L. (*O. majorana*) (Lamiaceae) is an aromatic Mediterranean plant widely used in food, cosmetics, and traditional medicine due to its aroma and rich content of bioactive compounds. While its leaves and flowers are commonly utilized, lignified stems are often discarded. This study compared hydroalcoholic extracts from the leaves and flowers (valuable fraction, VF) and stems (by-product, BP). Phytochemical analysis revealed qualitatively similar profiles, identifying 20 phenolic compounds, with Rosmarinic acid and Salvianolic acid B as the most and second most abundant, respectively. Antioxidant activity was evaluated in vitro using DPPH (IC_50_ [µg/mL]: VF 30.11 ± 3.46; BP 31.72 ± 1.46), H_2_O_2_ (IC_50_ [µg/mL]: VF 103.09 ± 4.97; BP 119.55 ± 10.58), and O_2_^•−^ (IC_50_ [µg/mL]: VF 0.71 ± 0.062; BP 0.79 ± 0.070). Both extracts (20 µg/mL) fully restored oxidative balance in hemin-stressed AC16 cardiomyocytes, without altering the expression of catalase, heme-oxygenase 1, superoxide dismutase 2, or ferritin. Anti-inflammatory activity in LPS-stimulated RAW 264.7 macrophages showed that VF (IC_50_ 400 µg/mL) reduced ^•^NO release to control levels, while BP achieved a ~60% reduction. Cytotoxicity was assessed on cancer cell lines: CaCo-2 (IC_50_ [µg/mL]: VF 154.1 ± 6.22; BP 305.2 ± 15.94), MCF-7 (IC_50_ [µg/mL]: VF 624.6 ± 10.27; BP 917.9 ± 9.87), and A549 (IC_50_ [µg/mL]: VF 720.8 ± 13.66; BP 920.2 ± 16.79), with no cytotoxicity on normal fibroblasts HFF-1 (IC_50_ > 1000 µg/mL for both extracts). Finally, both extracts slightly inhibited only CYP1A2 (IC_50_ [µg/mL]: VF 497.45 ± 9.64; BP 719.72 ± 11.37) and CYP2D6 (IC_50_ [µg/mL]: VF 637.15 ± 14.78, BP 588.70 ± 11.01). These results support the potential reuse of *O. majorana* stems as a sustainable source of bioactive compounds for nutraceutical and health-related applications.

## 1. Introduction

*O. majorana* L., commonly known as sweet marjoram, is a bushy perennial plant belonging to the Lamiaceae family and well distributed in most of the Mediterranean area, although native to Cyprus and Turkey [1]. This species is known by several scientific names, which are now considered homotypic synonyms, such as *Amaracus majorana* (L.) Schinz and Thell., *Majorana hortensis* Moench, *Majorana majorana* (L.) H.Karst., *Majorana vulgaris* Gray, *Origanum odorum* Salisb., and *Thymus majorana* (L.) Kuntze. In addition, in terms of taxonomy, *O. majorana* has been attributed about 23 heterotypic synonyms [1].

**Figure 1:**
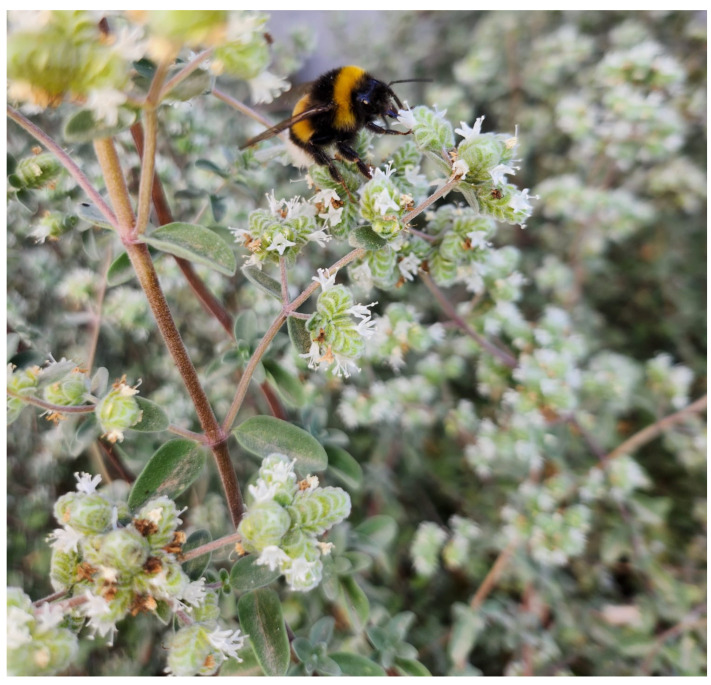
*Bombus terrestris* on *O. majorana* L. (Lamiaceae) flowers at the collection site. Syracuse, Italy, June 2022.

The plant can reach a maximum height of 60 cm and possesses small, simple, and hairy leaves that are oval to oblong in shape, with a characteristic aromatic scent due to the presence of essential oils [2]. The flowers are small, tubular, white or pale pink, hermaphrodite or female, with gray-green spike bracts, and the flowering period extends from May to September [2]. It grows best in well-drained, sandy, or loamy soils and prefers warm and sunny environments in areas with high levels of sunlight and moderate water availability, such as open grasslands, farmed fields, and dry, rocky habitats [3]. Because of its thickened leaf cuticles and trichomes, which reduce water loss, it is well-suited to drought conditions. The nectar-rich blossoms of *O. majorana* also attract pollinators such as bees and butterflies, which are beneficial to the ecosystem (Figure 1) [4]. Because this plant gives different insect species a place to live and food, it enhances biodiversity in Mediterranean habitats. Additionally, it can improve soil health and discourage some pests as a companion plant, which makes it useful in sustainable farming methods [5,6].

The food industry widely uses the dried leaves of sweet marjoram as flavoring agents for dressings and in the formulation of aromatized and fortified wines such as vermouth and bitters. *O. majorana* is a medicinal plant known for its use in traditional medicine. Different parts of this plant are utilized to treat several ailments, including cough, cramps, and acute diarrhea [7]. Several beneficial properties, including expectorant, carminative, antiseptic, antispasmodic, hepatoprotective, cardioprotective, antitumor effects, and notable anti-inflammatory and antioxidant activities, have been attributed to the plant [8].

*O. majorana* is well known for its distinctive aroma, which is due to the production of a well-characterized and chemically diverse essential oil with a broad spectrum of biological activities [7,9]. The essential oil is particularly rich in monoterpene hydrocarbons and their oxygenated derivatives (monoterpenoids), such as linalool, carvacrol, thymol, and borneol [9], which are highly appreciated in the cosmetic and perfumery industries [10,11]. Sesquiterpenes and sesquiterpenoids are also present, although typically in lower concentrations [9]. Traditionally, essential oil is extracted by hydro distillation from the leaves or the whole aerial parts of the plant [9], although more advanced extraction techniques, such as microwave-assisted extraction and supercritical CO_2_ extraction, have been successfully applied to improve yield and preserve thermolabile compounds [12,13].

It also contains various classes of phenolic compounds, including phenolic acids, flavonoids, and tannins. Notable compounds found in *O. majorana* extracts include carnosic acid, ferulic acid, caffeic acid, luteolin and its derivatives, apigenin and its derivatives, quercetin, and Rosmarinic acid [2]. These compounds are primarily responsible for the biological activities of the phenolic fraction of the plant [14].

With some exceptions, the leaves and flowers of this aromatic plant are the parts mostly used in all the commercial sectors listed above, while stems and the exhausted matrix from the distillation of essential oils represent by-products. Transforming by-products into new resources minimizes waste, reduces emissions, and optimizes raw materials, thereby promoting a more efficient and environmentally friendly circular economy model [15].

This study aimed to investigate whether the extract from stems of *O. majorana*, classified as a by-product (BP), can be valorized for nutraceutical and cosmeceutical purposes. Firstly, the BP was compared with the extract obtained from leaves and flowers (considered the valuable fraction, VF). The extractions were performed using a green, sustainable technique, and qualitative and quantitative phytochemical characterization was performed through the combined use of spectroscopic (UV/Vis-DAD) and spectrometric (MS) detectors coupled to high-performance liquid chromatography HPLC. Secondly, a comparison of the biological activities of both BP and VF extracts was carried out, focusing mainly on antioxidant properties, anti-inflammatory effects, inhibition of cytochrome isoforms, and cytotoxicity against cancer cells in different in vitro cell and cell-free models.

## 2. Results

### 2.1. Extraction Yield and Phytochemical Analyses by HPLC-ESI-MS of VF and BP from O. majorana

*O. majorana* aerial parts were homogenously collected in order to calculate accurately the percentage of waste. Specifically, flowering lignified stems of 56 ± 5 cm in length were harvested, and leaves and flowers (valuable fraction, VF) were manually separated from stems (by-product, BP). The BP percentage was calculated to be 40.11%. The extraction yields of VF and BP from *O. majorana*, expressed as the percentage of extract obtained per dry matrix, were 23.08% (VF) and 12.18% (BP), respectively. As expected, VF exhibited the highest extraction yield, whereas BP showed a yield of about half.

**Figure 2:**
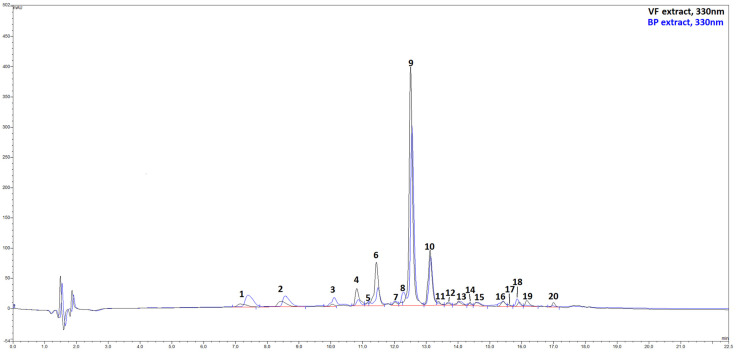
HPLC/DAD chromatograms, visualized at 330 nm of VF (black line) and BP (blue line) extracts from *O. majorana*. See text for further information and experimental details.

The qualitative and quantitative characterization of the extracts’ phenolic profile was carried out by HPLC/DAD and UPLC/ESI-MS. The combined use of liquid chromatography associated with spectroscopic and spectrometric detectors allows obtaining two sets of independent and complementary data that, together with an exhaustive literature search on identical or similar matrices, lead to the tentative identification of 20 different metabolites in the extracts (Figure 2, Figures S1 and S2). Chromatograms reported in Figure 2 show that VF and BP were nearly identical from a qualitative point of view, with Rosmarinic acid (peak 9), broadly considered as a marker for the Lamiaceae family, and salvianolic acid B (peak 10) as dominant compounds. The flavone luteolin, being present in the extract with five related compounds, is the most representative flavonoid in these matrices. A series of caffeic and p-coumaric acid derivatives, mainly with quinic acid, complete the compositional landscape. However, the extracts differed in the number of constituents. In particular, VF was found to be richer in phenolic compounds than BP, with polyphenol contents of 7926.4 mg/100 g of VF extract against 5688.8 mg/100 g of BP extract (Table 1). As mentioned, Rosmarinic acid (peak 9) was the most abundant compound in both extracts (VF: 3321.1 mg/100 g of extract; BP: 2152.9 mg/100 g of extract), followed by salvianolic acid B (peak 10) (VF: 1700.7 mg/100 g of extract; BP: 1219.5 mg/100 g of extract), luteolin-*O*-glucuronide (peak 6) (VF: 1176.4 mg/g of extract; BP: 409.0 mg/100 g of extract), and an apigenin derivative, apigenin di-*C*-hexoside (peak 2) (VF: 315.0 mg/100 g of extract; BP: 416.4 mg/100 g of extract).

### 2.2. In Vitro Cell-Free Antioxidant Properties of VF and BP from O. majorana

The antioxidant properties of VF and BP were initially tested using three reactive species, namely DPPH, superoxide anion, and hydrogen peroxide. Table 2 shows that the two extracts possessed strong and similar antioxidant activities in the three systems tested, with IC_50_ values of 30.11 µg/mL (VF) and 31.72 µg/mL (BP) for the DPPH test, 0.71 µg/mL (VF) and 0.79 µg/mL (BP) for the SOD-like activity assay, and 103.09 µg/mL (VF) and 119.55 µg/mL (BP) for the catalase-like activity assay.

### 2.3. MTT Test on Normal and Cancer Cells

**Figure 3:**
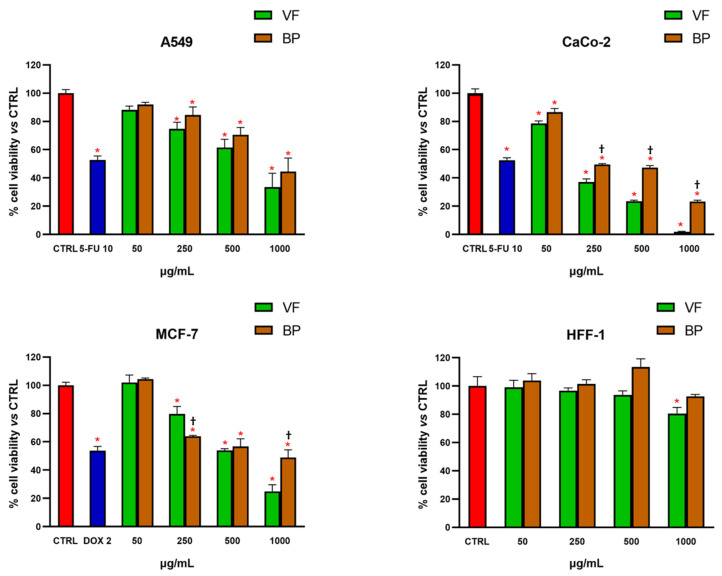
Effect of VF and BP hydroalcoholic extracts on cell viability in A549, CaCo-2, and MCF-7 cancer cell lines and HFF-1 normal cells. MTT tests were performed on the various cell lines treated with different concentrations of extract (from 50 to 1000 μg/mL) for 72 h. Data are represented as the means ± S.D. of three independent experiments. Confidence intervals were calculated by one-way ANOVA test: * Significant vs. untreated control cells; ^†^ BP significant vs. VF at the same concentration; *p* < 0.05.

The effect of VF and BP on cell viability was evaluated both in normal and cancer cell lines by the MTT test. As shown in Figure 3, treatment for 72 h resulted in significant and concentration-dependent reduction in cell viability against all tested cancer cell lines (A549, CaCo-2, and MCF-7), with CaCo-2 cells being the most sensitive to the treatments (Table 3). In contrast, the viability of the normal cell line HFF-1 was hardly affected by exposure to the extracts, and only a slight reduction in cell viability was observed after incubation with VF at the highest concentration tested (1000 µg/mL). These findings demonstrate that the extracts exert a selective reduction in cell viability toward cancer cells, supporting their safety in non-cancerous cells.

**Figure 4:**
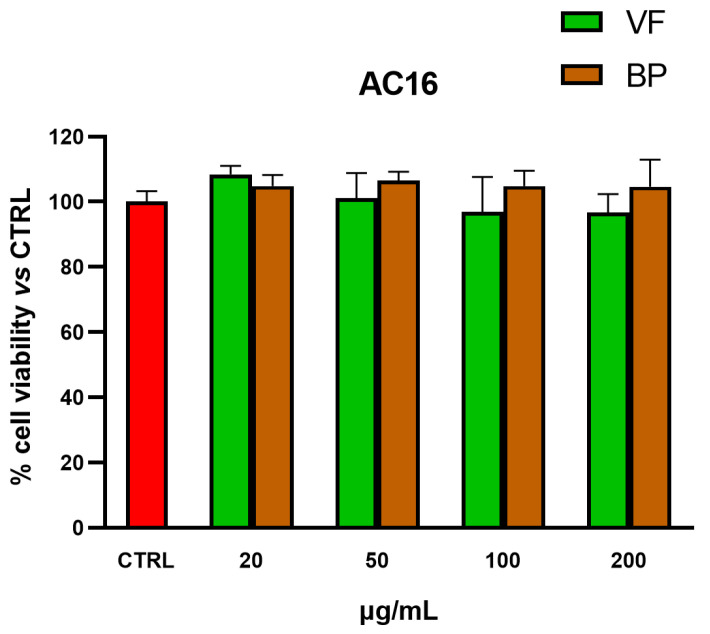
Cytotoxic effect of VF and BP hydroalcoholic extracts from *O. majorana* in AC16 cardiomyocytes. MTT tests were performed in AC16 cells treated with different concentrations of extract (from 20 to 200 μg/mL) for 48 h. Data are represented as the means ± S.D. of three independent experiments. Confidence intervals were calculated by one-way ANOVA test.

**Figure 5:**
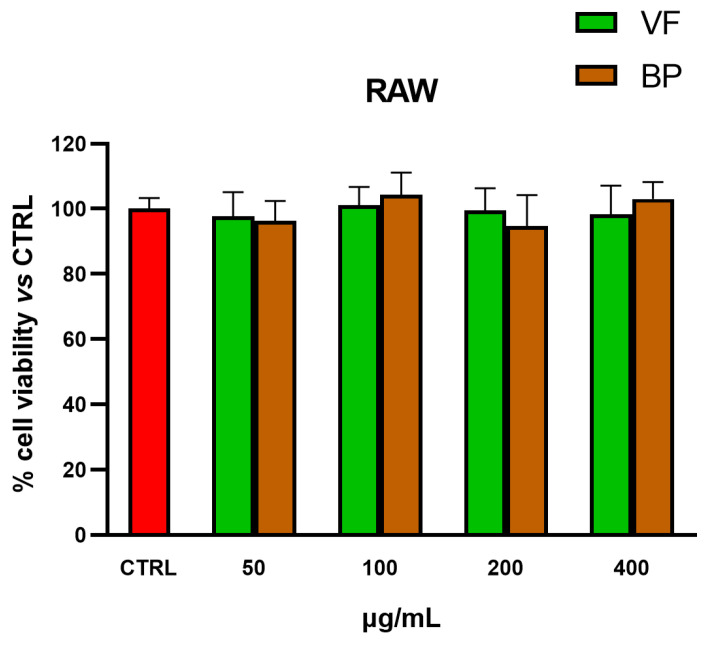
Cytotoxic effect of VF and BP hydroalcoholic extracts from *O. majorana* in RAW 264.7 macrophages. MTT tests were performed in RAW 264.7 cells treated with different concentrations of extract (from 50 to 400 μg/mL) for 24 h. Data are represented as the means ± S.D. of three independent experiments. Confidence intervals were calculated by one-way ANOVA test.

MTT tests were also used for selecting nontoxic extract concentrations for subsequent explorations in AC16 cardiomyocytes and RAW 264.7 macrophages. As shown in Figure 4 and Figure 5, none of the tested concentrations was found to be toxic, neither in AC16 (20-50-100-200 µg/mL for 48 h) nor in RAW 264.7 (50-100-200-400 µg/mL for 24 h) cells.

### 2.4. Antioxidant Activity in Cells

#### 2.4.1. Determination of ROS Levels in Human AC16 Cardiomyocytes

**Figure 6:**
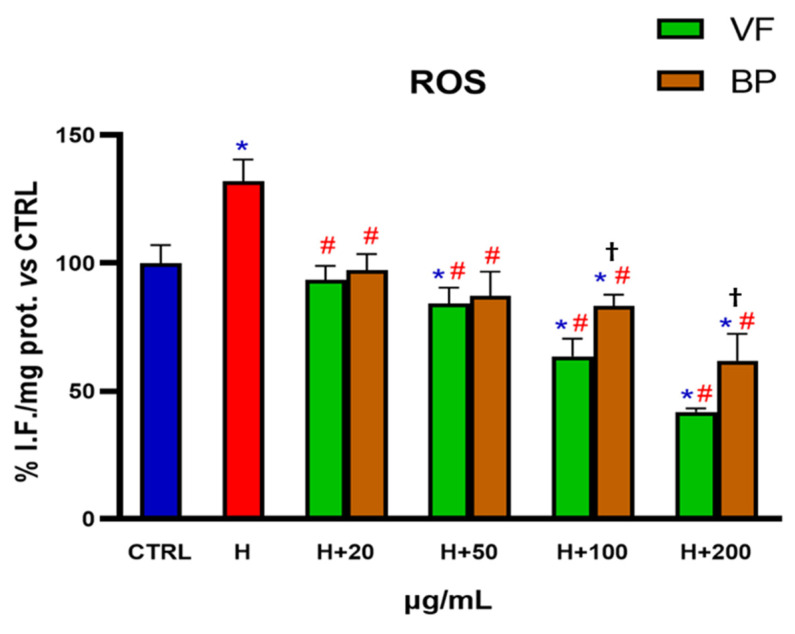
Antioxidant activity of VF or BP extracts from *O. majorana* in AC16 cardiomyocytes challenged with hemin. Untreated AC16 cells (CTRL) and cells pretreated with different concentrations of extracts were exposed to hemin (H, 1 µM). Each result represents the mean ± S.D. of three experiments. Confidence intervals were calculated by one-way ANOVA test. * Significant vs. non-stressed control cells; # significant vs. hemin-stressed cells; ^†^ BP significant vs. VF at the same concentration; *p* < 0.05.

AC16 cells challenged with hemin were used as a model of oxidative stress in order to assess if the antioxidant activity of the extracts, already established in in vitro cell-free systems, was reproducible in a cellular model. Specifically, AC16 cells were pretreated for 48 h with different concentrations of VF or BP (20-50-100-200 µg/mL) and then exposed for 6 h to 1 µM hemin to induce ROS production, thus simulating a condition of oxidative stress. As shown in Figure 6, even the lowest concentration of extracts tested (20 µg/mL) was able to lower intracellular ROS to levels found in control cells. Higher concentration of extracts further diminished hemin-induced ROS production in a concentration-dependent manner, with the VF extract being more powerful than the BP counterpart at the concentrations of 100 and 200 µg/mL. Interestingly, VP and BP reduced ROS levels to below control values when used at 100 and 200 µg/mL.

#### 2.4.2. Gene Expression of Antioxidant Enzymes in Human AC16 Cardiomyocytes

**Figure 7:**
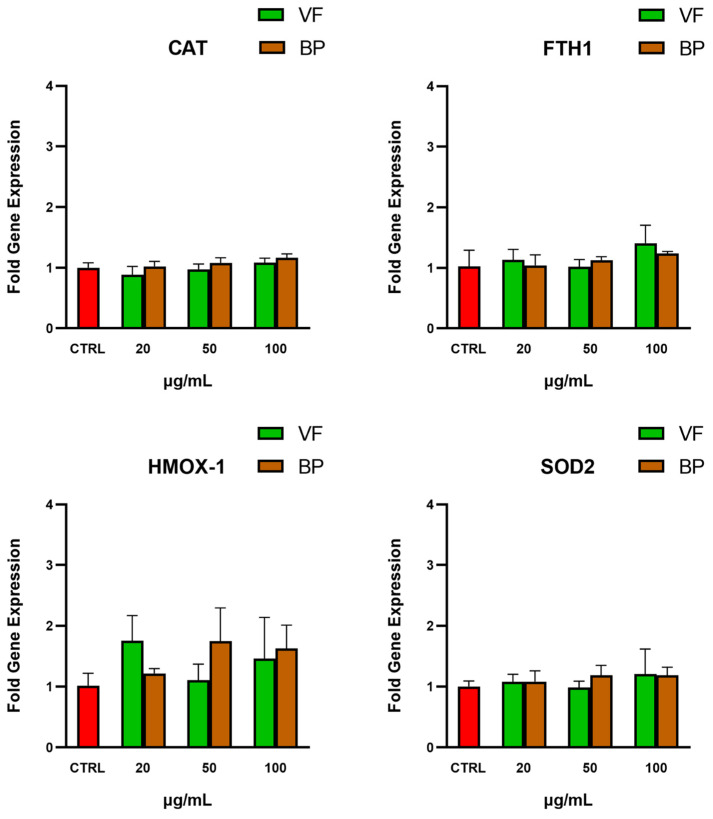
Gene expression levels of antioxidant genes in AC16 cardiomyocytes after exposure to the VP and BP extracts. qPCR analysis of relative gene expression of four endogenous antioxidant enzymes: heme oxygenase-1 (*HMOX-1*), ferritin heavy chain 1 (*FTH1*), mitochondrial superoxide dismutase (*SOD2*), and catalase (*CAT*) in untreated AC16 (CTRL), and cells pretreated with different concentrations of VF or BP extracts from *O. majorana.* Each result represents the mean ± S.D. of three experiments. Confidence intervals were calculated by one-way ANOVA test.

The potential indirect antioxidant activity of the extracts on AC16 cells was evaluated by analyzing the gene expression of four endogenous antioxidant genes, namely heme oxygenase-1 (*HMOX-1*), ferritin heavy chain 1 (*FTH1*), mitochondrial superoxide dismutase (*SOD2*), and catalase (*CAT*). As shown in Figure 7, incubation with the extracts did not significantly modify the expression of these genes, suggesting that the antioxidant activity of the extracts is primarily direct and does not involve modulation of the endogenous antioxidant systems examined.

### 2.5. Anti-Inflammatory Activity in Cells

#### Determination of ^•^NO Release in Murine RAW 264.7 Macrophages

**Figure 8:**
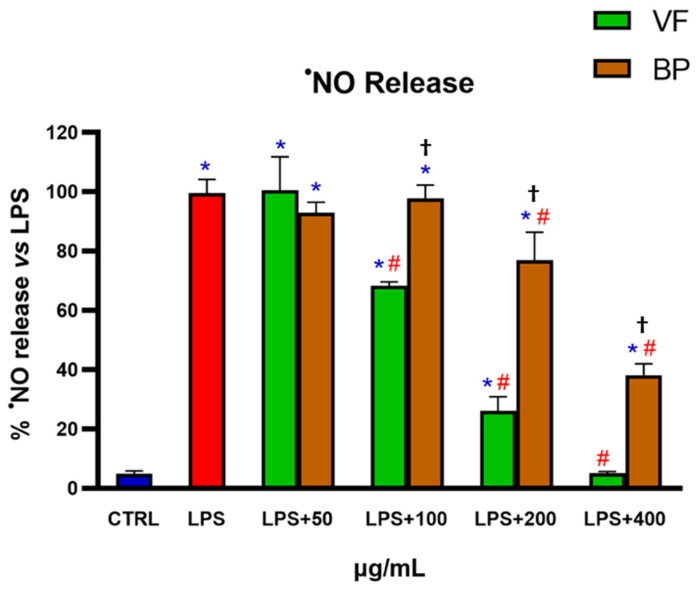
^•^NO released in RAW 264.7 macrophages challenged with LPS in the presence of VF or BP extracts from *O. majorana*. Untreated RAW 264.7 cells (CTRL), cells pretreated with different concentrations of VF or BP extracts, and/or cells activated with LPS (2 µg/mL) were used in these experiments. Each result represents the mean ± S.D. of four experiments. Confidence intervals were calculated by one-way ANOVA test. * Significant vs. non-stressed control cells; # significant vs. LPS-activated cells; ^†^ BP significant vs. VF at the same concentration; *p* < 0.05.

The anti-inflammatory effect of VF and BP was evaluated by using LPS-activated RAW 264.7 cells. Specifically, cells were pretreated with different concentrations (50-100-200-400 µg/mL) of extracts for 6 h, followed by stimulation with LPS (2 µg/mL) for 18 h. The results in Figure 8 show that both extracts exhibited a significant, concentration-dependent anti-inflammatory activity. VF was the most effective, reducing ^•^NO production to levels comparable to untreated control, thereby completely reversing the LPS-induced inflammatory response.

### 2.6. Inhibition of Cytocrome P450 Isoforms

The potential inhibitory effects of VF and BP on different cytochrome P450 isoforms were evaluated. No significant inhibition was observed for CYP3A4 and CYP2C19. However, the activities of CYP1A2 and CYP2D6 were affected at high doses, as indicated by the IC_50_ values shown in Table 4: 497.5 μg/mL (VF) and 719.7 μg/mL (BP) for CYP1A2, and 637.5 μg/mL (VF) and 588.7 μg/mL (BP) for CYP2D6.

## 3. Discussion

*O. majorana* is a medicinal and aromatic plant of the Lamiaceae family, well known not only for its essential oil rich in bioactive terpenoids, but also for its high content of polyphenols, which contribute significantly to its broad spectrum of biological activities. Among these, Rosmarinic acid, salvianolic acids, and various flavonoids, including luteolin and apigenin derivatives, stand out for their potent antioxidant, anti-inflammatory, and cytoprotective effects. While most studies have focused on the leaves and aerial parts, other plant fractions, such as lignified stems, remain poorly explored, despite their significant contribution to biomass and potential as sources of valuable phytochemicals. In this study, we investigated and compared the phenolic composition of hydroalcoholic extracts from the VF and BP of *O. majorana* in order to valorize lignified stems as a possible source of bioactive compounds, which represent 40,11% of the total collected biomass. Specifically, we investigated biological activities related to chemoprevention, oxidative stress, inflammation, and drug–botanical interactions. The goal was to explore the BP fraction for potential nutraceutical and cosmeceutical applications in line with sustainable resource utilization and circular economy principles. Given the potential for industrial application and the scalability of the extraction technique, we chose digestion under continuous stirring. This method is well-suited for industrial environments and provides high extraction efficiency [16]. A hydroalcoholic solution was selected as the solvent due to its high effectiveness in extracting phenolic compounds [16]. Additionally, ethanol is regarded as a renewable resource that is biodegradable and has minimal environmental and health impacts [17,18,19]. It is also easy to use in industrial applications. The effectiveness of this extraction technique was confirmed by the phytochemical analysis conducted by HPLC-ESI-MS. The findings revealed that both VF and BP extracts exhibited a rich and qualitatively similar phenolic profile, with differences primarily in the abundance of compounds. VF showed a significantly higher total polyphenol content than BP, particularly in key constituents such as Rosmarinic acid and salvianolic acid B. These results align with the existing literature highlighting *O. majorana* as a rich source of phenolic compounds with potential biological effects [2]. The glycosides identified, luteolin-O-glucuronide and apigenin di-C-hexoside, have previously been reported as components of *O. majorana*-derived extracts [20,21]. Rosmarinic acid has also been identified as the most abundant compound in other studies [22,23], further supporting *O. majorana* as a reliable source of this compound. Importantly, our results suggest that BP is a valuable source of bioactive compounds with a phytochemical profile similar to that of VF, differing only quantitatively, with approximately 30% lower compound levels compared to the higher levels found in VF. However, to our knowledge, *O. majorana* stems remain an underexplored plant matrix, and no studies have yet been conducted on their qualitative and quantitative phenolic characterization, as most research on this species has focused on the leaves, flowers, or whole aerial parts.

The antioxidant activities of plant extracts play a crucial role in protecting the body from the harmful effects of free radicals, particularly ROS, which can cause oxidative damage to cells, proteins, lipids, and DNA [24]. By counteracting these hazardous substances, antioxidants aid in lowering oxidative stress, which is linked to several chronic diseases, including neurological disorders, cancer, heart disease, and aging [25]. Secondary metabolites of plant extracts have potential therapeutic effects for treating or preventing oxidative damage-related disorders and promoting general health [26,27]. In cell-free tests that targeted reactive species such as hydrogen peroxide, superoxide anion, and DPPH, BP demonstrated antioxidant properties similar to VF, despite having a lower polyphenol content. These findings imply that BP retains a substantial portion of the plant’s antioxidant potential even though VF has a higher polyphenol content.

Additionally, both extracts showed dose-dependent reductions in ROS levels in hemin-stressed AC16 cardiomyocytes. The model was chosen to evaluate the preventive efficacy of the extracts against a traumatic event, which could result in the release of the pro-oxidant free heme and consequent oxidative stress [28,29,30]. Interestingly, in our experimental model, there was no change in the gene expression of the four antioxidant proteins examined, suggesting that the extracts primarily work by directly scavenging radicals rather than by upregulating endogenous antioxidant defenses. Our data are in line with previous studies reporting strong antioxidant activities of *O. majorana* extracts in different models of oxidative stress, both in vitro and in vivo [2,31].

Natural compounds are extensively studied for their capacity to protect cells from harmful conditions and to prevent various human diseases, including cancer [32]. Extracts from *O. majorana* were demonstrated to inhibit carcinogenesis and oncogenic mutations and to exert antiproliferative and apoptotic activities in different cancer cell lines [33,34]. Our results on the reduction in cell viability of both extracts further support their potential use in preventing cancer progression. VF and BP phytocomplexes exhibited selective cytotoxicity toward cancer cell lines, particularly CaCo-2. At the same time, no significant effect was recorded on normal cells such as HFF-1 up to a concentration of almost 1000 µg/mL, indicating that, overall, extracts from *O. majorana* are safe [2,22,35].

As part of our ongoing comparison analysis, we looked at the two *O. majorana* extracts potential anti-inflammatory properties. Inflammation is the immune system’s natural response to infection and sterile injury and protects the body by eliminating pathogens and repairing damaged tissue. However, when it becomes chronic or uncontrolled, it represents a risk factor for numerous diseases. Inflammation is often linked to an unhealthy lifestyle, stress, obesity, pollution, or autoimmune diseases. This type of low-level inflammation can damage tissues over time, contributing to the development of diseases better known as non-communicable or lifestyle diseases [36]. Modern science focuses on controlling chronic inflammation through nutrition, physical exercise, and stress management [37,38]. In addition, many natural compounds can positively modulate low chronic systemic inflammation [39,40]. In our study, VF and BP extracts significantly reduced the release of ^•^NO induced by LPS in RAW 264.7 macrophages, with VF demonstrating superior efficacy. These results are consistent with existing literature reporting the notable anti-inflammatory activity of *O. majorana* extracts [23,41]. In particular, a similar outcome was observed in a study conducted on RAW 264.7 macrophages stimulated with 1 μg/mL LPS, where a complete inhibition of ^•^NO production was achieved with 200 μg/mL of *O. majorana* hydroalcoholic aerial part extract [42]. The higher concentration required in the present study (400 μg/mL) to reduce NO release to control levels can likely be attributed to the use of a higher LPS concentration (2 μg/mL). These findings highlight the potential health benefits of *O. majorana* in conditions related to inflammation and suggest that its by-products may also have valuable bioactivities, although to a slightly lesser extent.

These results are coherent with the existing literature about the composition and biological activities of the single constituents present in our extracts. Rosmarinic acid, the most abundant in both VF and BP, possesses a variety of well-established, health-promoting activities, including antioxidant, anti-inflammatory, and anticancer properties. Specifically, the anticancer activity has been assessed in vitro, in different cancer cell lines, including breast and colorectal cancer cells, and in vivo, in rats with induced colon cancer [43,44]. Salvianolic acid B, the second most present compound in both extracts, together with a series of luteolin-derivatives, particularly abundant in both, have also been reported to be able to exert important biological activities, supporting our findings regarding the antioxidant, anti-inflammatory, and anticancer effects exerted by VF and BP [45,46,47].

To complete the comparison and exploration of the potential biological activities of the VF and BP extracts of *O. majorana*, we evaluated the possible inhibitory activity of the two phytocomplexes on the CYP450 isoforms. The inhibition of these enzymes by botanicals is crucial because it can lead to drug-botanical interactions, potentially altering the bioavailability and efficacy of medications. These interactions can result in decreased drug metabolism, leading to increased drug levels and toxicity, or vice versa, causing therapeutic failure [48]. Preliminary data on CYP450 isoform inhibition revealed that both extracts could partially inhibit CYP1A2 and CYP2D6 at high concentrations while showing no significant activity against CYP3A4 and CYP2C19. Although not indicative of strong drug interaction potential at typical nutraceutical concentrations, these results warrant further pharmacokinetic investigation, particularly if the extracts are to be included in complex formulations or used alongside pharmaceuticals.

## 4. Materials and Methods

### 4.1. Chemicals and Reagents

Analytic-grade organic solvents, including dimethyl sulfoxide (DMSO), 2′,7′-dichlorofluorescein diacetate (DCFH-DA), and 2,2-diphenyl-1-picrylhydrazyl (DPPH), were sourced from VWR (Milan, Italy). All other substances, unless indicated otherwise, were obtained from Sigma-Aldrich (Milan, Italy). Additionally, unless stated otherwise, all materials and media utilized for cell culture were procured from ThermoFisher Scientific in Milan, Italy. HPLC grade solvents, water, and acetonitrile were obtained from Carlo Erba (Milan, Italy). High-purity standard chlorogenic acid (used to quantify caffeic acid derivatives), p-coumaric acid (used to quantify the corresponding derivatives), Rosmarinic acid, Salvianolic acid B, luteolin (which was used to quantify all luteolin derivatives), and apigenin (used to quantify apigenin derivatives and acacetin) were all purchased from Sigma-Aldrich (VWR Italia, Milan, Italy).

### 4.2. Plant Material and Extraction Procedure

The aerial parts (56 ± 5 cm length) of *O. majorana* L. were collected during the flowering time in early June (2022). All vegetal material (1000 g), constituted by leaves and flowers (598.9 g) and lignified stems (401.1 g), was homogeneously sampled from different plants, which grew almost wild in a private organic garden in Syracuse (Sicily, Italy). The identification of the botanical sample was performed by one of the authors (G.A.M.). A voucher specimen (06/22) was deposited in the Department of Drug and Health Sciences, University of Catania. After harvesting, the plant material was gently washed and wiped and immediately dried in a ventilated oven for three days at 35 ± 2 °C. Then, leaves and flowers were manually separated from stems and stored at room temperature in amber glass jars before use.

Two different extracts were prepared respectively from leaves and flowers (valuable fraction, VF) and from stems (by-product, BP) by digestion under continuous stirring using a hydroalcoholic solution with 50% *v*/*v* of ethanol as solvent, with the time of extraction being 4 h, temperature of extraction 40 ± 5 °C, and plant matrix/solvent ratio (g of matrix/mL of solvent) 1:30. The extraction procedure was repeated three times to exhaust the matrix. Then, the extracts were filtered, dried using a rotary evaporator (Rotavapor Büchi R110, BUCHI, Flawil, Switzerland), redissolved in water, and dried by freeze-drying. The dried extracts were stored at −80 °C before use.

### 4.3. HPLC/DAD and HPLC/-ESI-MS Analyses

HPLC/DAD analyses were carried out as reported by Luca et al. [49]. The HPLC instrument (Ultimate3000) was equipped with a binary high-pressure pump, an automated sample injector, and a thermostated column compartment (Thermo Scientific, Milan, Italy). The analysis was carried out using a reverse-phase column (Gemini C18, 250 × 4.6 mm, 5 μm particle size; Phenomenex, Torrance, CA, USA) equipped with a guard column (Gemini C18 4 × 3.0 mm, 5 μm particle size; Phenomenex). The data were analyzed with the software Chromeleon Chromatography Information Management System (v. 6.80).

Chromatograms were registered at 254, 280, 330, and 350 nm to detect all polyphenol subclasses. Quantification of cinnamic acids and polyphenols was performed via Uv-vis/DAD employing external reference standards possessing an identical or similar chromophore. Accordingly, quantification of quercetin derivatives was performed at 350 nm using rutin (R^2^ = 0.9998) as reference control, whereas luteolin derivatives and luteolin were quantified using luteolin as external standard (R^2^ = 0.9997) at the same wavelength. Caffeoyl derivatives were quantified at 330 nm, making use of a calibration curve established with chlorogenic acid (R^2^ = 0.9998), whilst p-coumaroyl derivatives were quantified at 330 nm using p-coumaric acid (R^2^ = 0.9994). Quantification of Rosmarinic acid and salvianolic acid B was performed by using the corresponding commercial standards (R^2^ = 0.9997 and R^2^ = 0.9995, respectively); apigenin was quantified using itself in the form of a high-purity standard (R^2^ = 0.9999). Finally, quantification of flavanone hesperetin was accomplished at 280 nm using the corresponding pure reference (R^2^ = 0.9995). Each result represents the mean ± S.D. of three determinations.

To unambiguously identify the chromatographic signals and/or to confirm peak assignments, UPLC/ESI/MS analyses were also performed on the extracts employing a Vanquish UPLC System equipped with a quaternary high-pressure pump F (VF-P20-A), a photodiode array detector (VC-D11-A), a thermostated column compartment and an automated sample injector (VF-A10-A) (Thermo Fisher Scientific, Inc., Milan, Italy) coupled to a TSQ Fortis Plus Mass spectrometer. Collected data were processed through the software Thermos Scientific Xcalibur version 4.5. The chromatographic method discussed above was adapted to a reverse-phase 10 cm column (Luna Omega C18, 100 × 2.1 mm, 1,6 μm particle size, Phenomenex Italia s.r.l., Bologna, Italy); while the MS method has been set with a scan-range (*m*/*z*) of 150–1500 in and negative (2200 V) mode, Q1 resolution of 0.7 (FWHM) and 20 eV as source fragmentation; the ion source type was H-ESI with a static spray voltage.

### 4.4. Determination of Antioxidant Activity by DPPH Test

The assay was carried out as reported by Luca et al. [49], with some modifications. Briefly, 1 mL of an ethanolic solution with different concentrations of extract (10-25-50-75-100 µg/mL) or ascorbic acid, used as a reference compound (final concentrations 1-2.5-5-7.5-10 µg/mL), and 86 µM (final concentration) 1,1-Diphenyl-2-picrylhydrazyl (DPPH) was incubated for 10 min in darkness. Control samples consisted of an 86 µM DPPH solution in ethanol. Then, the absorbance was measured at λ = 517 nm by a spectrophotometer, Hitachi UV 2000 (Hitachi, Tokyo, Japan), and the results were expressed as a percentage of absorbance decrease with respect to the control. Results are presented as IC_50_ mean ± S.D. of three experiments.

### 4.5. SOD-like Activity Assay

The assay was carried out as reported by Genovese et al. [50], with some modifications. Briefly, 10 μL of aqueous extract solution (final concentrations 0.25-0.5-1-2.5-5 µg/mL) or ascorbic acid, used as a reference compound (final concentrations 0.010-0.015-0.020-0.025-0.030 µg/mL), were added to 990 μL of a reaction mixture for the in vitro production of superoxide anion (O_2_^•−^), prepared by mixing 100 mM triethanolamine–diethanolamine buffer, pH 7.4, 3 mM NADH, 25 mM/12.5 mM EDTA/MnCl_2_, and 10 mM β-mercaptoethanol. Control samples consisted of the same reaction mixture without extract or reference compound, plus 10 μL of water. After 20 min of incubation at room temperature in the dark, the decrement of NADH absorbance was measured for 5 min at λ = 340 nm by a spectrophotometer, Hitachi UV 2000, and the results were expressed as a percentage reduction of NADH oxidation. Results are presented as IC_50_ mean ± S.D. of three experiments.

### 4.6. Catalase-like Activity Assay

The assay was performed as reported by Al-Amiery et al. [51], with some modifications. Briefly, 10 μL of aqueous extract solution (final concentrations 25-50-100-200-300 µg/mL) or ascorbic acid solution, used as reference compound (final concentrations 10-25-50-75-100 µg/mL), at different concentrations, were added to 990 μL of 24 mM H_2_O_2_ solution in phosphate buffer (pH 7.4). Control samples consisted of 990 μL of 24 mM H_2_O_2_ in phosphate buffer (pH 7.4) plus 10 μL of water. After 10 min of incubation at room temperature in the dark, the absorbance was measured at λ = 240 nm, and the results were expressed as a percentage of absorbance decrease with respect to the control. Results are presented as IC_50_ mean ± S.D. of three experiments.

### 4.7. Cell Culture

CaCo-2 (human colon adenocarcinoma, ATCC number HTB-37^TM^) and MCF-7 (human breast adenocarcinoma, ATCC number HTB-22^TM^) cell lines were maintained in Minimum Essential Medium (MEM, Gibco 11095-080, Thermo Fisher Scientific, Waltham, MA, USA) containing 1 mM pyruvate, 2 mM Glutamine, 100 U/mL penicillin, 100 μg/mL streptomycin, 1% Non-Essential Amino Acids (NEAA) and 10% Fetal Bovine Serum (FBS, Gibco A5256701). A549 (human lung carcinoma ATCC number CCL-185^TM^), RAW 264.7 (murine macrophages ATCC number TIB-71^TM^), and HFF-1 (human fibroblast, ATCC number SCRC-1041^TM^) cell lines were cultured in Dulbecco’s Modified Eagle Medium (DMEM, Gibco 41966-029) high glucose (4.5 g/L) containing 1 mM pyruvate, 4 mM Glutamine, 100 U/mL penicillin, 100 μg/mL streptomycin, and 10% FBS. AC 16 (human cardiomyocytes, ATCC number CRL-3568^TM^) cell line was cultured in DMEM/F-12 (Gibco 11320-033) containing 17.5 mM glucose, 2.5 mM glutamine, 0.5 mM pyruvate, 2.438 g/L sodium bicarbonate, 10% Newborn Calf Serum (NBCS, Gibco 16010-159), and 100 U/mL penicillin, 100 μg/mL streptomycin. Cells were kept in a humidified atmosphere with 5% CO_2_ at 37 °C. When ~80% confluence was reached, cells were rinsed with PBS without Ca^2+^/Mg^2+^ and harvested with 0.25% trypsin-EDTA solution (Gibco 25200-056). Then, trypsin was inactivated by adding three times its volume of medium, the suspension was centrifuged at 1000 rpm for 5 min, and the cell pellet was resuspended in fresh complete medium. The cells were then expanded in flasks or seeded for performing the following experiments.

### 4.8. Cell Viability by MTT Assay

To assess the effects of the extracts on cell viability, the MTT test was applied as reported by Tomasello et al. [52], with some modifications. Briefly, cells were seeded in 96-well plates at a density of 10 × 10^3^ (A549, CaCo-2, HFF-1, MCF-7, RAW 264.7) or 5 × 10^3^ (AC16) cells/well. After 24 h, the cells were treated with different concentrations of extract (10–1000 µg/mL) for 24, and/or 48, and/or 72 h. At the end of the treatment, the medium was replaced with 0.5 mg/mL MTT medium solution for 1 h. Then, the medium was removed, and 100 μL of DMSO was added to each well. When the formazan was completely dissolved, absorbance was measured at λ = 570 nm using a microplate reader, Sinergy HT Biotek. Then, 5-fluorouracil and doxorubicin were used as reference compounds (A549, CaCo-2, MCF-7). Results are expressed as a percentage of cell viability vs. untreated control cells and represent the mean ± S.D. of four experiments.

### 4.9. Reactive Oxygen Species Assay

AC16 cells were seeded at a density of 2 × 10^4^ cells/well in 24-well plates. After 24 h, cells were treated with extracts at different concentrations (20-50-100-200 μg/mL) for 48 h prior to challenge with the pro-oxidant molecule hemin (1 μM) for 6 h. At the end of the incubation, the levels of intracellular ROS were measured following the procedure reported by Tomasello et al. [52], using the dichlorodihydrofluorescein diacetate (H2DCF-DA) method, with some modifications. The H2DCF-DA probe was added at a 5 μM final concentration, and cells were incubated at 37 °C for 30 min. Then, each well was washed twice with cold PBS without Ca^2+^/Mg^2+^, and cells were lysed with 250 μL/well of a 2.5 mg/mL digitonin solution for 1 h at 4 °C. Finally, cell lysates were centrifuged at 12 × 10^3^ g for 10 min at 4 °C, and 100 μL of supernatant was used to measure intensity of fluorescence (I.F.) by microplate reader Sinergy HT Biotecn (λex = 488 nm/λem = 525 nm). Results are expressed as a percentage of I.F./mg of protein vs. untreated control cells and represent the mean ± S.D. of three experiments.

### 4.10. Quantification of ^•^NO Release

The ^•^NO release quantification was carried out as reported by Malfa et al. [53], with some modifications. RAW 264.7 cells were seeded at a density of 5 × 10^4^ cells/well in 96-well plates. After 48 h, cells were treated with different extract concentrations (50, 100, 200, and 400 μg/mL) for 6 h prior to stimulation with 2 μg/mL lipopolysaccharides (LPS) for 18 h, still in the presence of the extract. At the end of the treatment, 100 μL were collected from each well and used for the quantification of nitrite (derived from ^•^NO oxidation) by adding the same volume of Griess reagent. After 30 min incubation at room temperature in the dark, the absorbance at λ = 546 nm was measured by a microplate reader, Sinergy HT Biotecn. Results are expressed as a percentage of ^•^NO released vs. LPS-activated cells and represent the mean ± S.D. of four experiments.

### 4.11. RNA Extraction and Gene Expression Evaluation by RT-qPCR

AC16 cells were seeded at a cell density of 10 × 10^4^ cells/well in 6-well plates. After 24 h, cells were treated with extracts at different concentrations (20-50-100 μg/mL), and after 24 h, cells were collected for RNA extraction by QIAGEN RNeasy^®^ Plus Mini Kit, following the procedure reported by the manufacturer. RNA was quantified by Thermo Scientific NanoDrop^TM^ One, and the retrotranscription procedure was performed by Thermo Scientific High-Capacity cDNA Reverse Transcription Kit, following the procedure reported by the manufacturer. Then, samples were stored at −20 °C before performing qPCR analysis.

The qPCR reaction mix was prepared using the Thermo Scientific TaqMan^TM^ Universal Master Mix II, with UNG, and TaqMan^TM^ primers for heme oxygenase 1 (*HMOX1*, Hs01110250_m1), mitochondrial superoxide dismutase (*SOD2*, Hs00167309_m1), ferritin heavy chain 1 (*FTH1*, Hs01694011_s1), and catalase (*CAT*, Hs00156308_m1). Hypoxanthine phosphoribosyltransferase 1 (*HPRT1*, Hs02800695_m1) was used as a housekeeping gene. All the reagents were used following the manufacturer’s instructions. The relative gene expression was calculated by the delta–delta Ct method. Each result represents the mean ± S.D. of three experiments.

### 4.12. Cytochrome P450 Isoforms Activity Assay

The potential inhibitory activity of VF and BP on different CYP450 enzyme isoforms (1A2, 3A4, 2C19, 2D6) was screened by fluorometric tests using the Abcam inhibitors screening Kits, respectively, ab211075, ab211077, ab211073, ab211079, following the manufacturer’s instructions. Briefly, in an opaque white 96-well plate, 20 µL of extract assay buffer solution (final concentrations 50-125-250-500-1000 µg/mL) was added to 50 µL of CYP enzyme solution. After 10 min of incubation at 37 °C, 30 µL of substrate/NADP+ reaction mix was added to each well to reach a final volume of 100 µL. Finally, the intensity of fluorescence was recorded by a microplate reader, Sinergy HT Biotek, in kinetic mode. Also, α-naphthoflavone (CYP1A2), ketoconazole (CYP3A4), ticlopidine (CYP2C19), and quinidine (CYP2D6) were used as positive inhibition controls (PICs). Each result represents mean ± S.D. of three experiments.

### 4.13. Statistical Analysis

Statistical analyses were performed by one-way ANOVA coupled with Tukey’s multiple comparison test. IC_50_ values were compared by *t*-test. *p* < 0.05 was considered significant.

## 5. Conclusions

This study compares the phytochemical profiles and a variety of bioactivities of the hydroalcoholic extracts from the VF and BP of *O. majorana*, focusing on chemoprevention, antioxidant defence, inflammation control, and possible drug-botanical interactions. Our findings support the sustainable exploitation of *O. majorana* by-products within a circular economy framework. Specifically, the two extracts exhibit nearly identical biological activities, differing only in their strength in specific cellular models. This could be attributed to the minor content of phenolic compounds, as evidenced by HPLC-ESI-MS analysis. However, considering the substantial similarity between the two phytochemical profiles, it is theoretically possible to obtain a quantitatively and qualitatively similar product from the two matrices by applying industrial processes for extract purification and enrichment. Therefore, we suggest the recovery of *O. majorana* stems as a valuable raw material for obtaining bio-active compounds beneficial for nutraceutical and/or phytotherapeutic applications. Although our study provides a detailed investigation of extracts obtained from *O. majorana* aerial parts, further studies using samples from different harvest years and cultivation origins will be necessary to validate and generalize the present findings.

## Figures and Tables

**Table 1:** Phenolic compounds in VF (valuable fraction) and BP (by-product) extracts from *O. majorana*. Results presented are the mean of three replicates (n = 3) and are expressed as mg/100 g of extract.

Peak	Rt (min) ^a^	Compound Tentative Identification	VF	BP
1	7.147	Caffeoyl-hexose	103.7 ± 5.9	298.9 ± 10.7
2	8.436	Apigenin di-*C*-hexoside	315.0 ± 4.5	416.4 ± 5.7
3	10.048	Luteolin di-*O*-glucuronide	63.4 ± 2.3	239.9 ± 1.7
4	10.815	Caffeoylquinic acid	291.1 ± 5.5	67.8 ± 5.2
5	11.146	Quercetin-*O*-glucuronide	45.4 ± 2.8	63.6 ± 4.3
6	11.431	Luteolin -*O*- glucuronide	1176.4 ± 9.0	409.0 ± 5.6
7	12.024	*p*-Coumaroylquinic acid 1	90.7 ± 5.6	79.1 ± 7.0
8	12.210	*p*-Coumaroylquinic acid 2	56.1 ± 2.1	277.0 ± 5.0
9	12.506	Rosmarinic acid ^b^	3321.1 ±45.9	2152.9 ± 19.0
10	13.118	Salvianolic acid B ^b^	1700.7 ± 45.0	1219.5 ± 7.0
11	13.382	Quercetin-O-hexoside	63.7 ± 2.0	19.7 ± 0.2
12	13.667	Dicaffeoylquinic acid 1	53.9 ± 3.4	n.d.
13	14.015	di-*p*-Coumaroylquinic acid 1	176.5 ± 1.3	138.4 ± 8.7
14	14.370	Dicaffeoylquinic acid 2	50.9 ± 4.8	17.3 ± 0.5
15	14.562	Luteolin ^b^	52.1 ± 2.5	38.2 ± 0.9
16	15.421	Methyluteolin	72.6 ± 4.3	60.3 ± 5.1
17	15.601	di-*p*-Coumaroylquinic acid 2	40.7 ± 2.3	25.7 ± 0.3
18	15.927	Salvianolic acid B isomer	44.0 ± 0.9	n.d.
19	16.162	Dimethylluteolin	107.2 ± 2.5	29.0 ± 0.8
20	17.001	Hesperetin ^b^	100.9 ± 1.2	16.3 ± 1.0
		Total Polyphenols	7926.4 ± 69.4	5688.8 ± 29.2

^a^ as the mean of three replicates; ^b^ identified with the help of the corresponding high-purity analytical commercial standard.

**Table 2:** In vitro cell-free antioxidant activities of VF, BP, and ascorbic acid (AA).

*O. majorana*	VF (IC_50_ μg/mL ± S.D.)	BP (IC_50_ μg/mL ± S.D.)	AA (IC_50_ μg/mL ± S.D.)
DPPH	30.11 ± 3.46	31.72 ± 1.46	4.97 ± 0.062
SOD-Like	0.71 ± 0.062	0.79 ± 0.070	0.019 ± 0.0055
Catalase-Like	103.09 ± 4.97	119.55 ± 10.58	42.55 ± 0.65

**Table 3:** Effects of VF and BP on cell viability. ^†^ BP Significant vs. VF; *p* < 0.05.

Cell Lines	VF (IC_50_ μg/mL ± S.D.)	BP (IC_50_ μg/mL ± S.D.)
CaCo-2	154.1 ± 6.22	305.2 ± 15.94 ^†^
MCF-7	624.6 ± 10.27	917.9 ± 9.87 ^†^
A549	720.8 ± 13.66	920.2 ± 16.79 ^†^
HFF-1	N.D. ^a^	N.D. ^a^

^a^ not detectable at the maximum concentration tested, 1000 µg/mL.

**Table 4:** Potential inhibitory effect of VF and BP on different CYP450 isoforms. ^†^ BP Significant vs. VF; *p* < 0.05.

CYP Isoform	VF (IC_50_ μg/mL ± S.D.)	BP (IC_50_ μg/mL ± S.D.)	PIC (IC_50_ μg/mL ± S.D.)
CYP1A2	497.45 ± 9.64	719.72 ± 11.37 ^†^	0.0404 ± 0.0021
CYP3A4	N.D. ^a^	N.D. ^a^	0.0563 ± 0.0040
CYP2C19	N.D. ^a^	N.D. ^a^	0.153 ± 0.0071
CYP2D6	637.15 ± 14.78	588.70 ± 11.01 ^†^	0.00630 ± 0.00046

^a^ not detectable at the maximum concentration tested, 1000 µg/mL.

## Data Availability

Data were generated at the Department of Drug and Health Science, University of Catania, and at the Faculty of Health, University Paris-Est Créteil-Inserm U955. Data supporting the results of this study are available from the corresponding authors on request.

## Supplementary Materials

The following supporting information can be downloaded at: https://www.mdpi.com/article/10.3390/plants14152264/s1, Figure S1. TIC chromatogram (mass range 150–1500 *m*/*z*), acquired in negative ion mode, of the VF extract object of this study. Uv-vis trace, visualized at 330 nm, is given for comparison purposes. Figure S2. mass spectra (mass range 150–1000 *m*/*z*, base peak visualization mode) for compounds **1**–**20** reported in Figure 2 and Table 2 in the text.

## Abbreviations

The following abbreviations are used in this manuscript:

AA	Ascorbic acid
BP	By-product
CAT	Catalase
CYP450	Cytochrome P450
DPPH	1,1-Diphenyl-2-picrylhydrazyl
FBS	Fetal Bovine Serum
FTH1	ferritin heavy chain 1
H2DCF-DA	dichlorodihydrofluorescein diacetate
HMOX1	heme oxygenase 1
I.F.	Intensity of fluorescence
ROS	Reactive oxygen species
S.D.	Standard Deviation
SOD	Superoxide dismutase
VF	Valuable fraction
