# Supplementary material for: Origanum majorana Extracts: A Preliminary Comparative Study on Phytochemical Profiles and Bioactive Properties of Valuable Fraction and By-Product
Source: Plants (Basel). 2025 Jul 23;14(15):2264. doi: 10.3390/plants14152264 (PMC12348732; doi:10.3390/plants14152264)

Supplementary: "*Origanum majorana* Extracts: A Preliminary Comparative Study on Phytochemical Profiles and Bioactive Properties of Valuable Fraction and By-product"

Figure S1. TIC chromatogram (mass range 150-1500m/z), acquired in negative ion mode, of the VF extract object of this study. Uv-vis trace, visualized at 330nm, is given for comparison purposes.

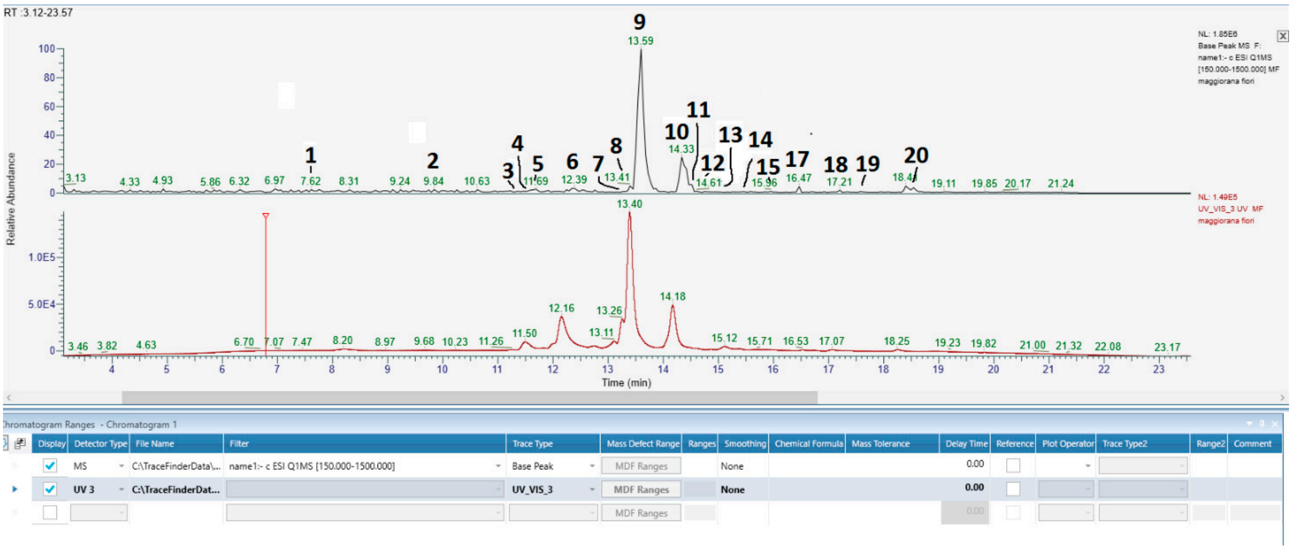

Figure S2: mass spectra (mass range 150-1000 m/z, base peak visualization mode) for compounds 1-20 reported in figure 2 and table 2 in the text. MW exact values from https://www.sisweb.com/referenc/tools/exactmass.htm

Peak 1, tentatively identified as caffeoyl-hexose, MW 342.095; diagnostic ion $[M-H]^-$.

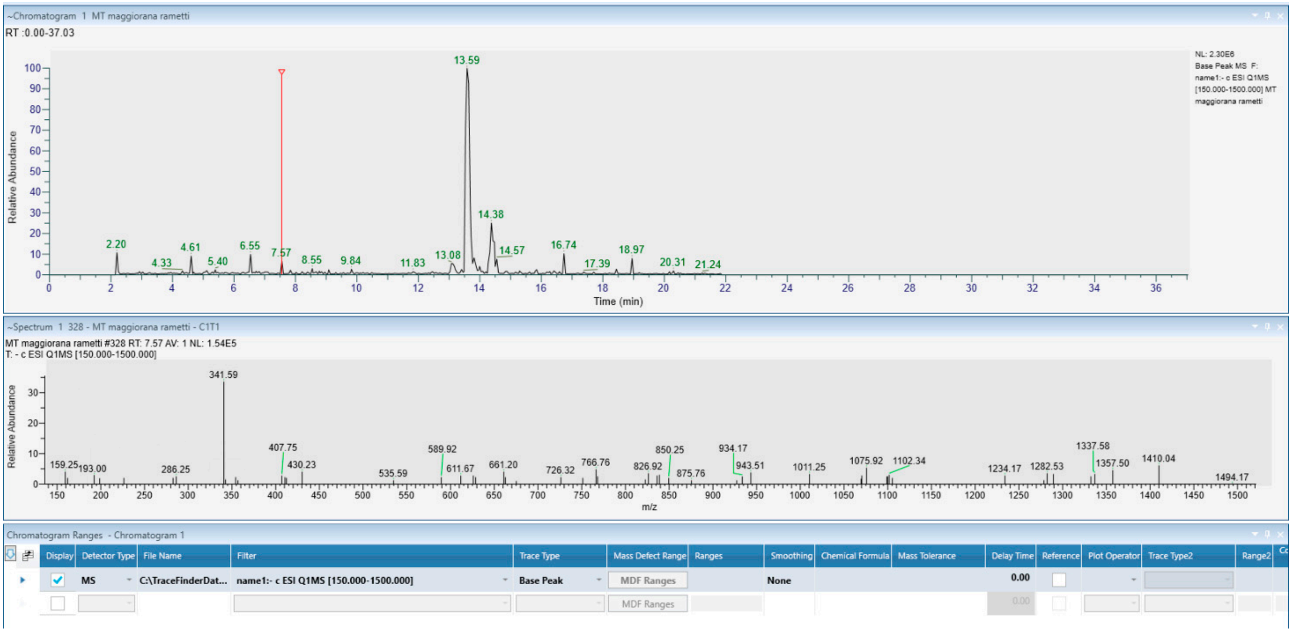

Peak 2, tentatively identified as apigenin di-C-hexoside, MW = 594,1584; diagnostic ion [M-H]$^-$. Identification of this compound was corroborated with our previous study on vicenin- 2 (apigenin 6,8 di-*C*-glucoside).

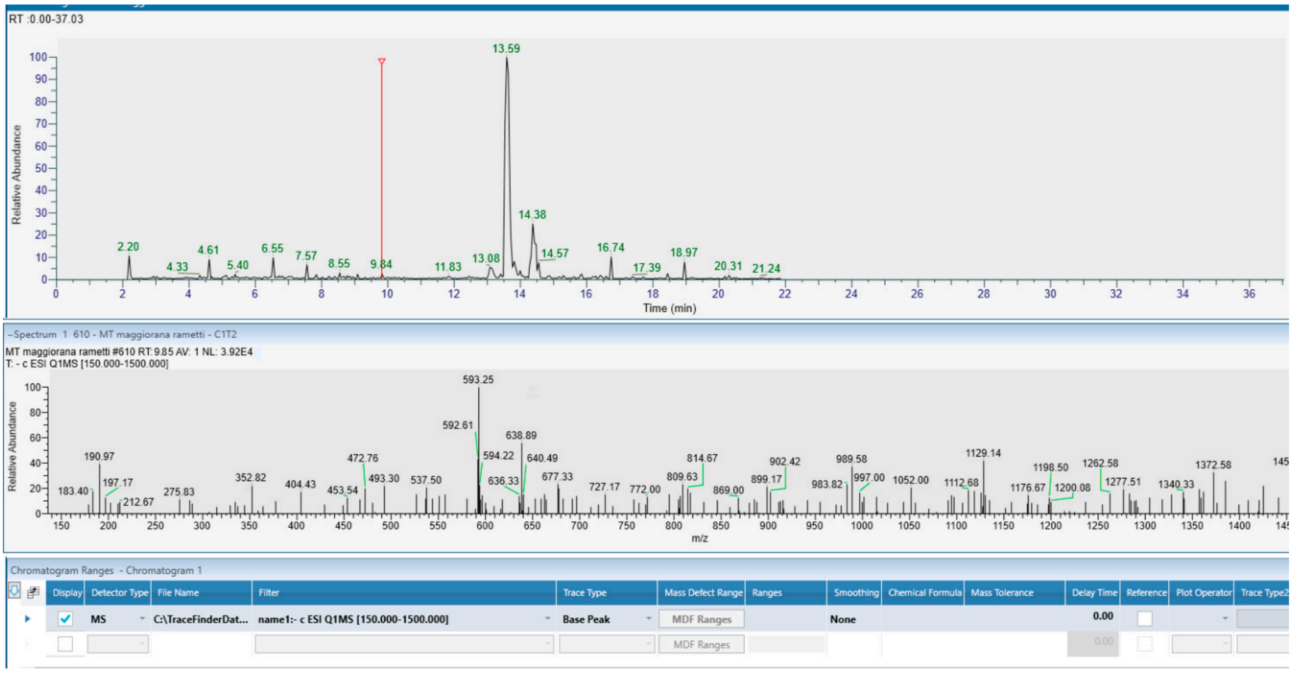

Peak 3, tentatively identified as luteolin di-O-glucuronide, MW = 638,1119; diagnostic ion [M-H+Na]$^-$

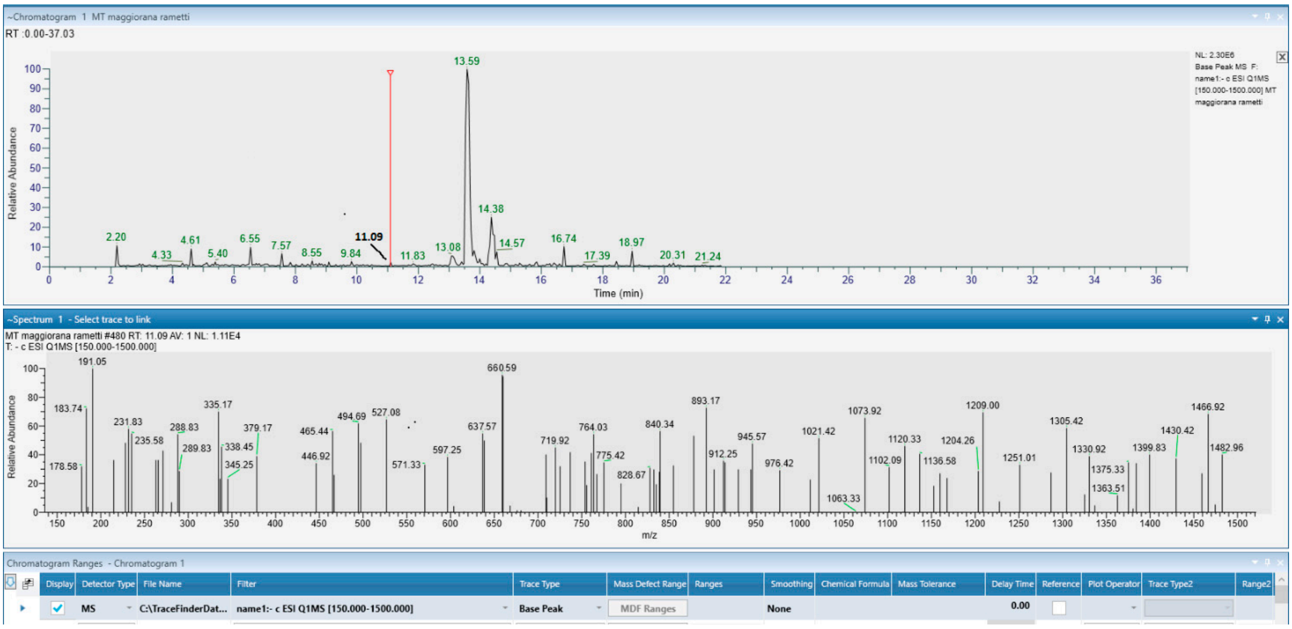

Peak 4, tentatively identified as caffeoylquinic acid (chlorogenic acid isomer), MW = 354,05; diagnostic ions $[M\text{-}H]^-$, 190.96 m/z (quinate ion)

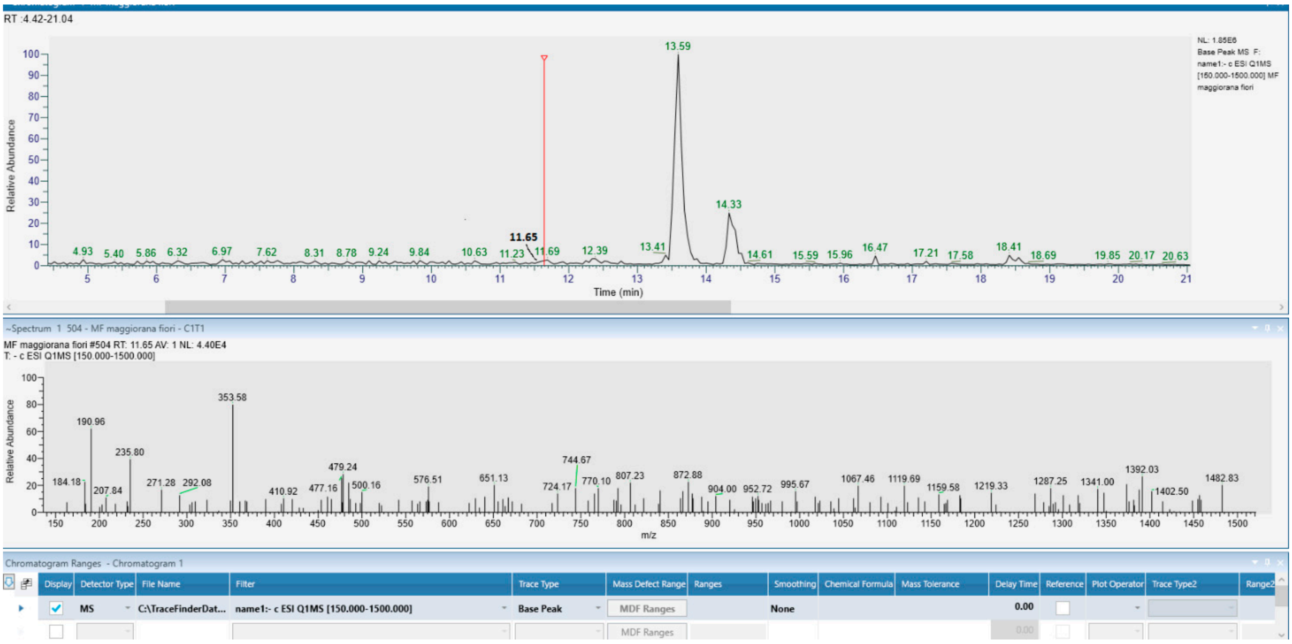

Peak 5, tentatively identified as quercetin-O-glucuronide, MW = 478,0747; diagnostic ion $[M\text{-}H]^-$

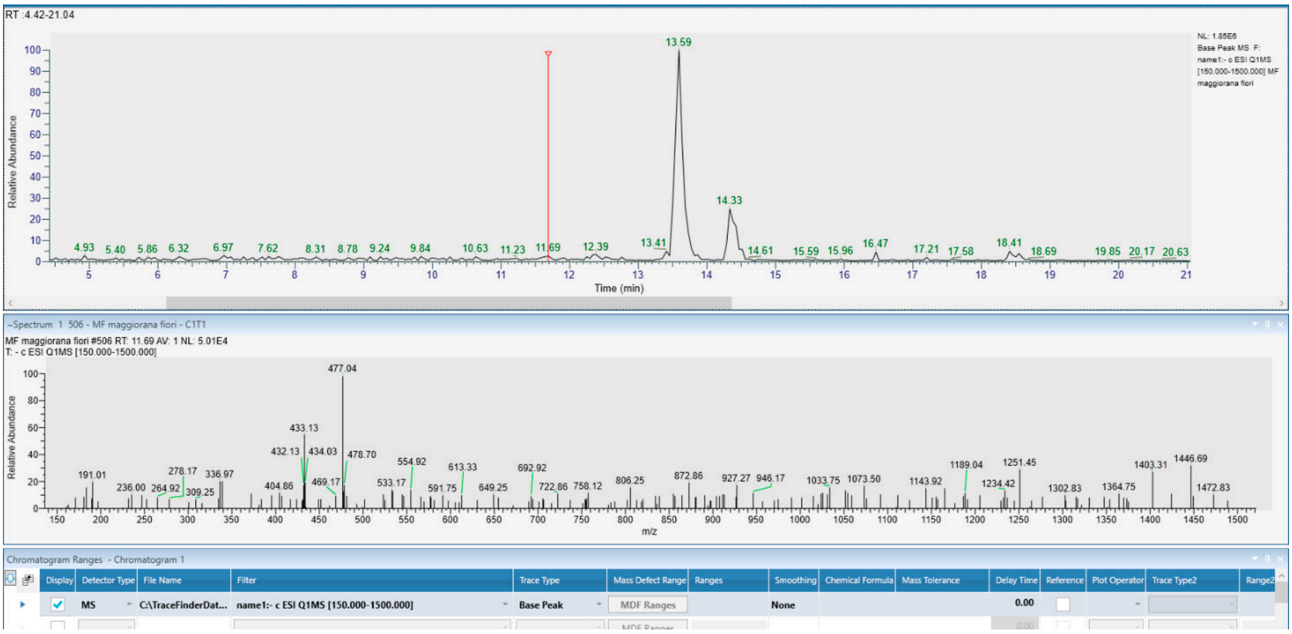

Peak 6, tentatively identified as luteolin-O-glucuronide, MW = 462,0962; diagnostic ion $[M-H]^-$

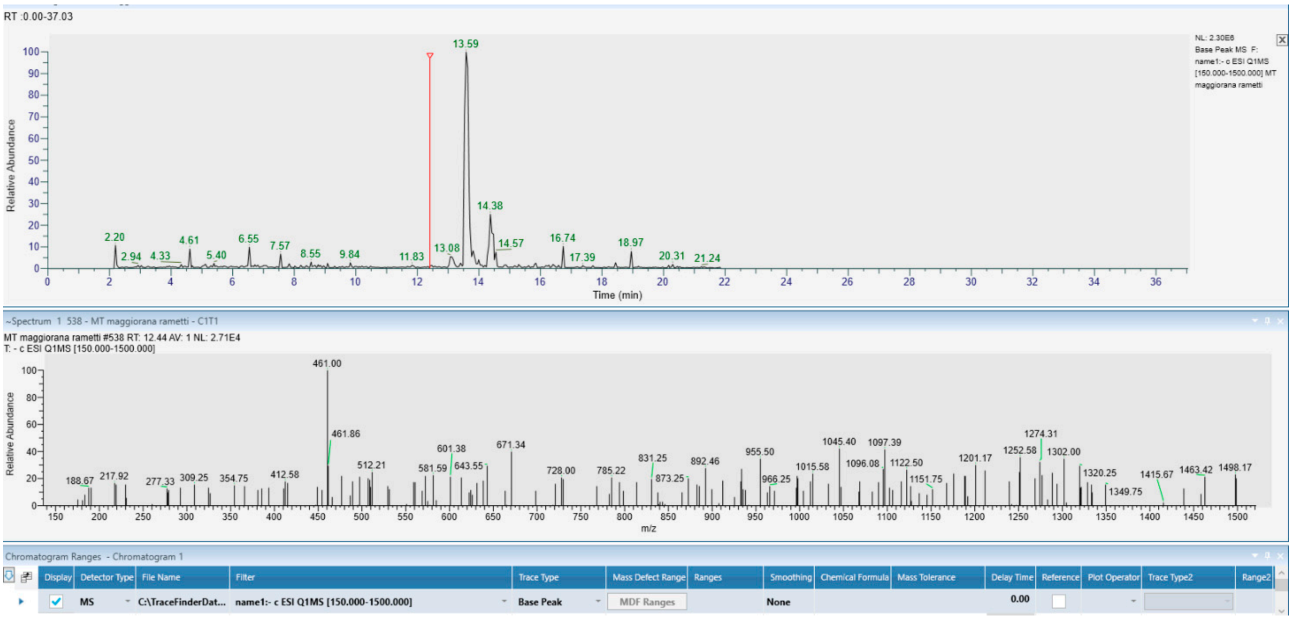

Peak 7-8, tentatively identified as two isomers of p-coumaroylquinic acid, MW = 338,1001; diagnostic ion $[M-H]^-$ and 190.87 m/z (quinate ion). Peaks 7 and 8 showed identical mass spectra

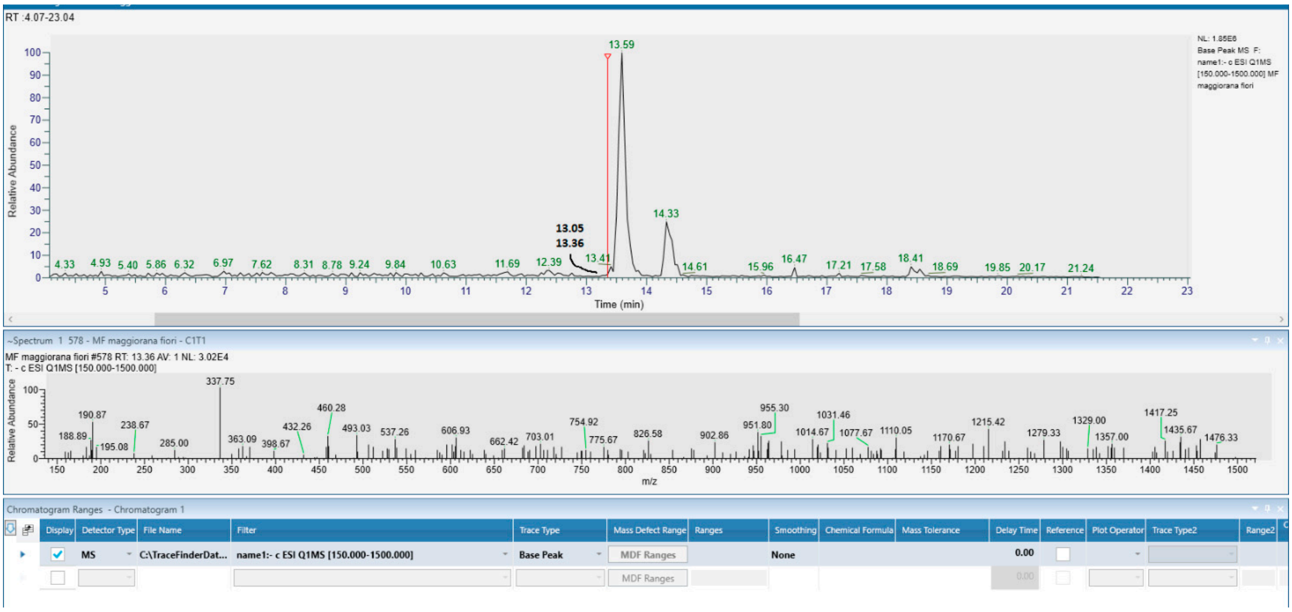

Peak 9, rosmarinic acid, MW = 360,0804; diagnostic ion [M-H]$^-$

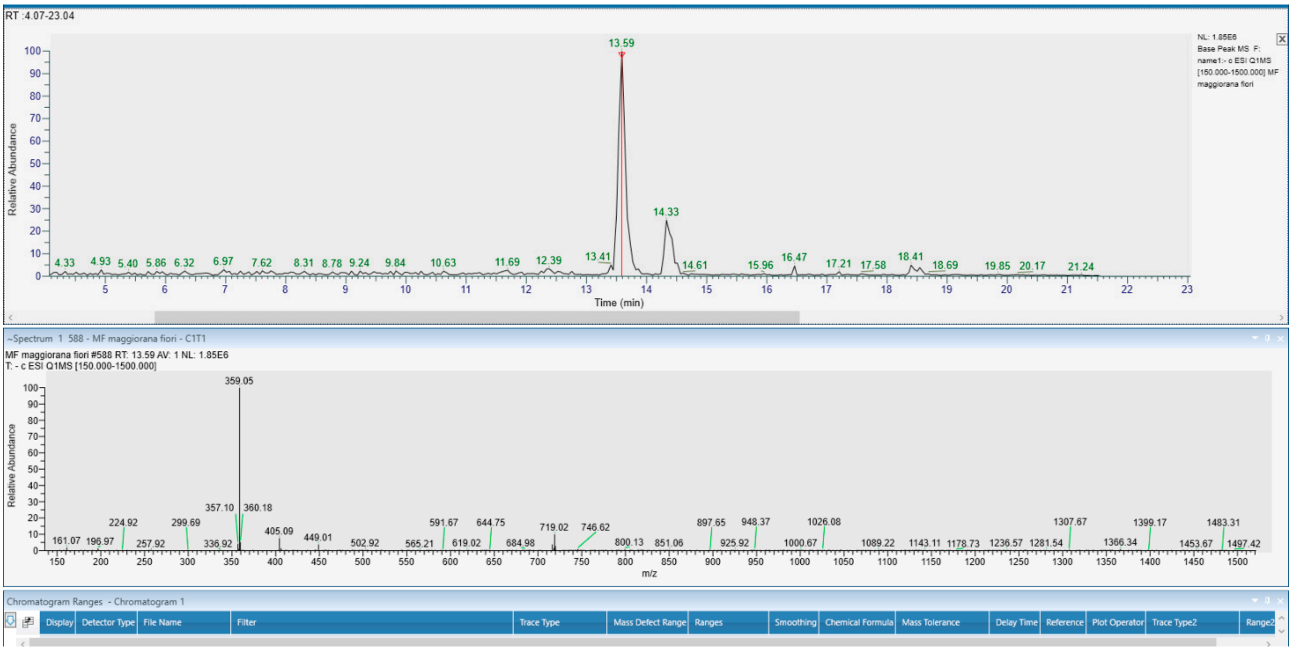

Peak 10, salvianolic acid B, MW = 718,1533; diagnostic ion [M-H]$^-$

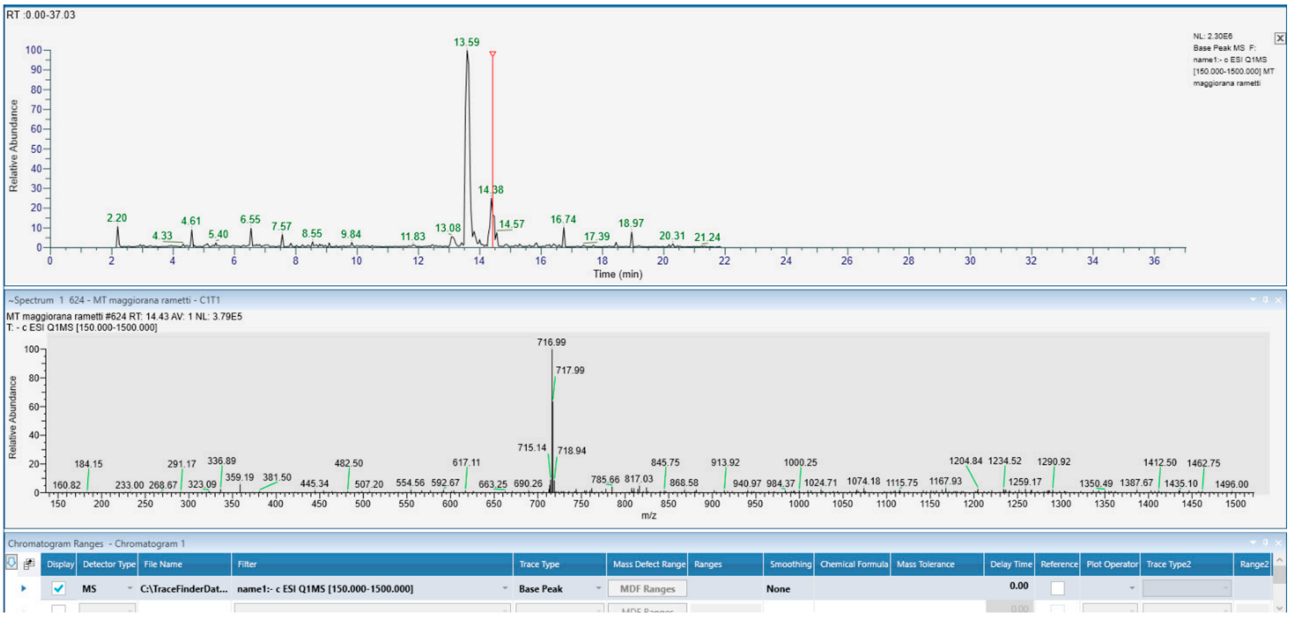

Peak 11, tentatively identified as quercetin-O-hexoside (quercetin 3-O-glucoside isomer), MW = 464,0954; diagnostic ion [M-H]$^-$

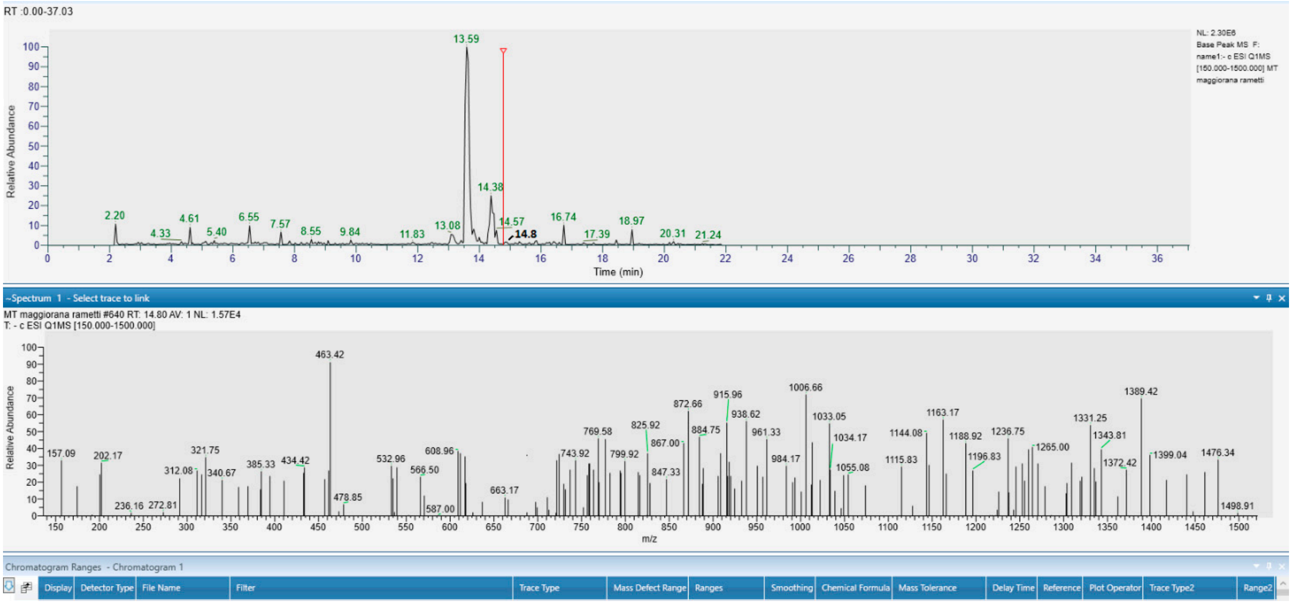

Peak 12, tentatively identified as di-caffeoylquinic acid isomer 1(cynarin isomer) MW = 516,1267; diagnostic ion [M-H]$^-$ and 190.96 m/z (quinate ion)

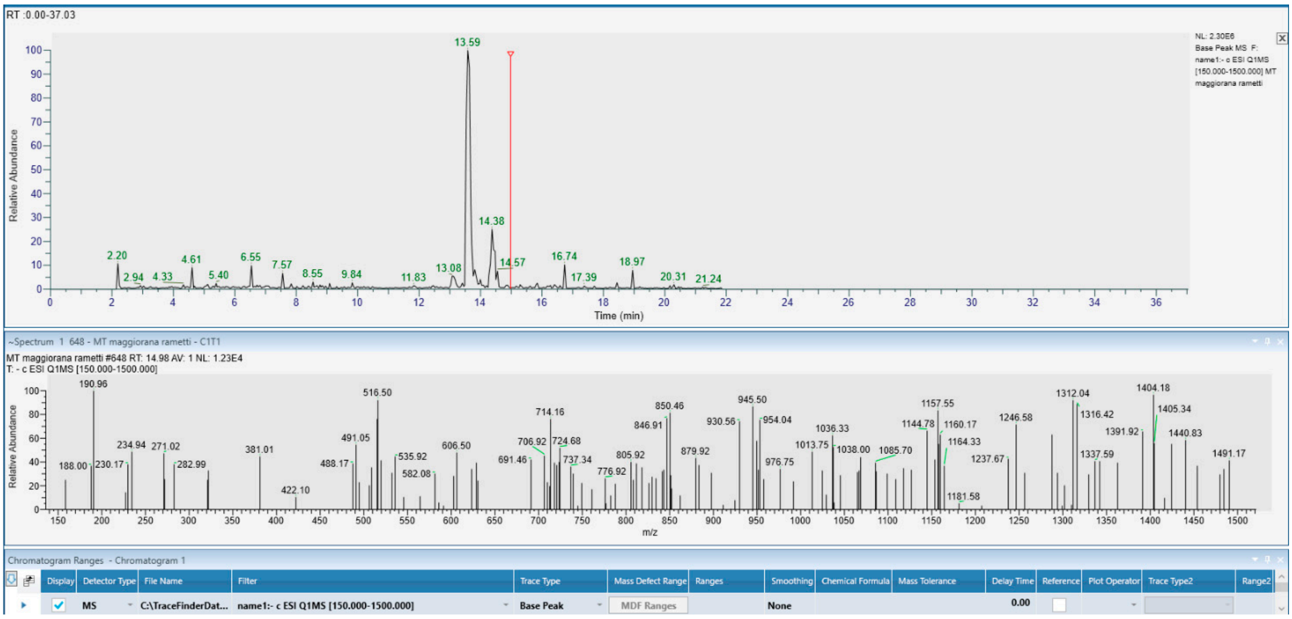

Peak 13, tentatively identified as di-p-coumaroylquinic acid 1, MW = 484,1369; diagnostic ion $[M-H]^-$ and 190.76 m/z (quinate ion)

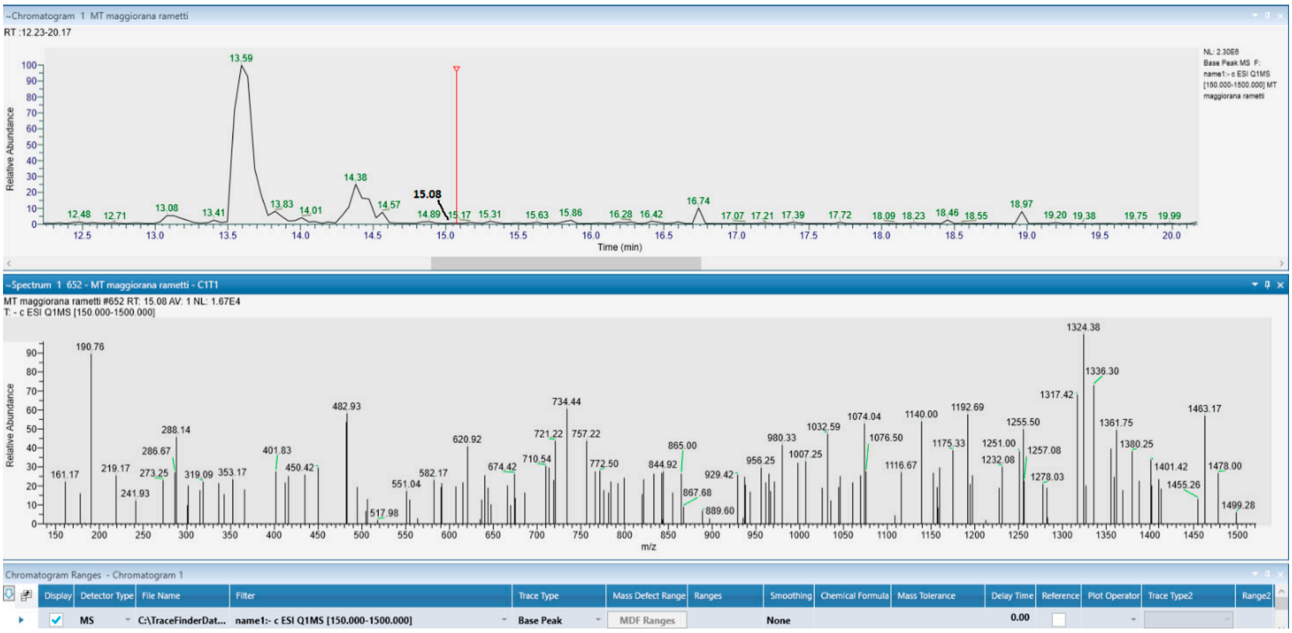

Peak 14, tentatively identified as di-caffeoylquinic acid isomer 2(cynarin isomer) MW = 516,1267; diagnostic ion $[M-H]^-$ and quinate ion

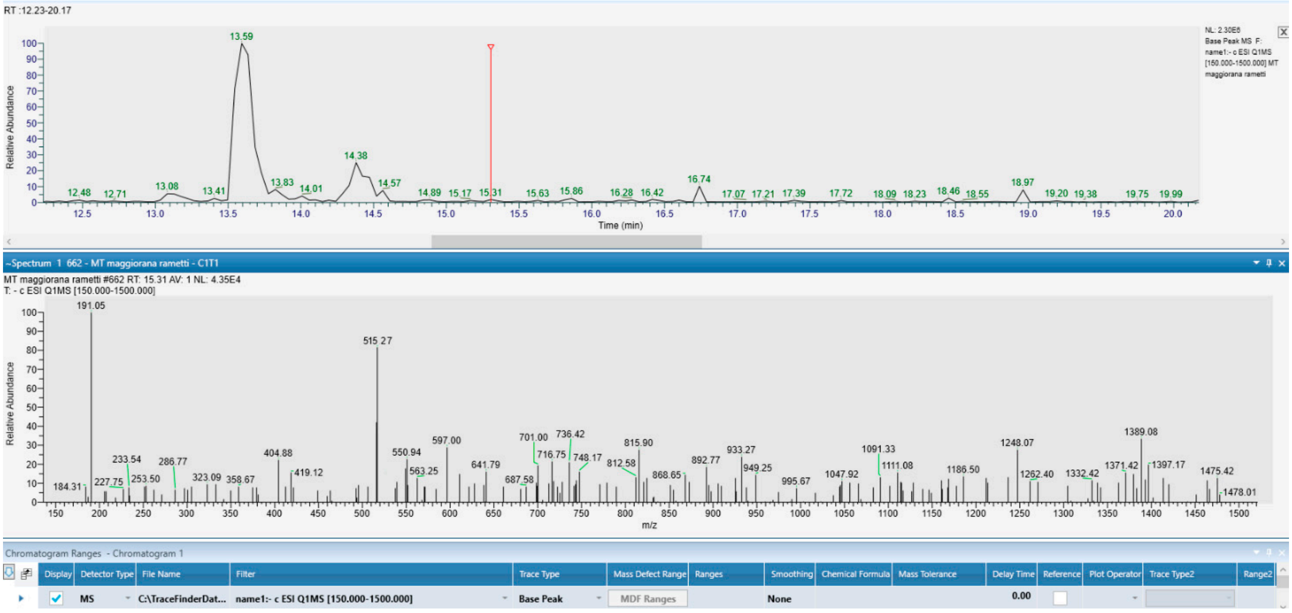

Peak 15, luteolin, MW=286,0477; diagnostic ion [M-H]$^-$

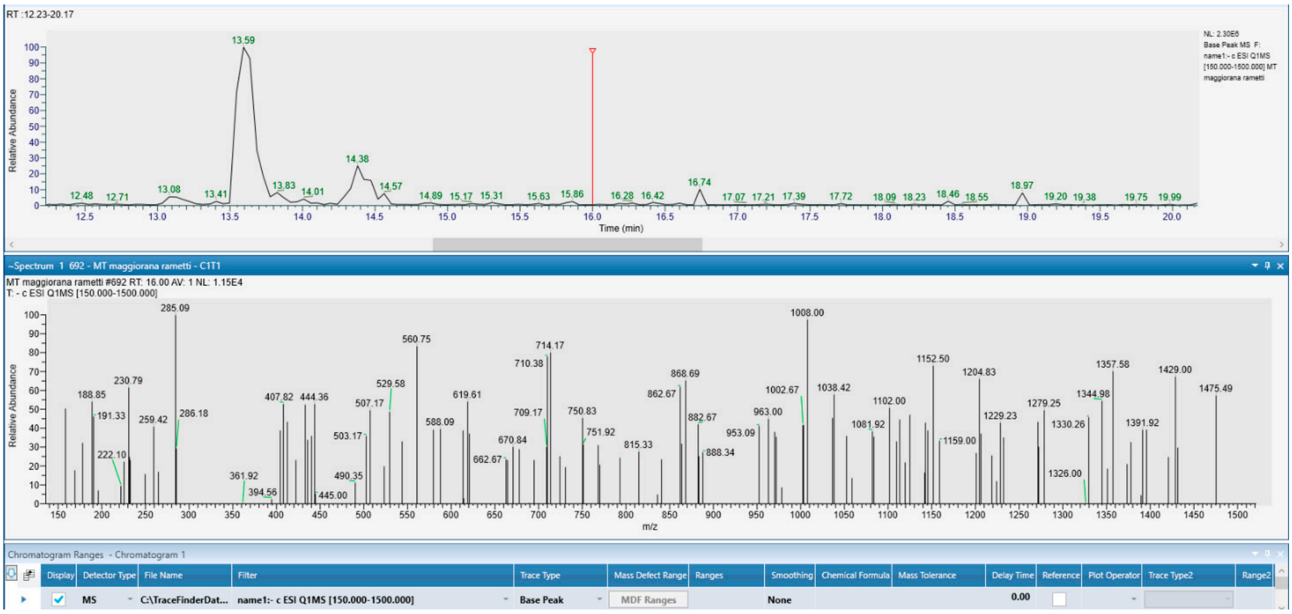

Peak 16, tentatively identified as methyl luteolin, MW = 300,0633; diagnostic ions 285.07 [M-H]$^-$ and 322.75 m/z [M-H+Na]$^-$

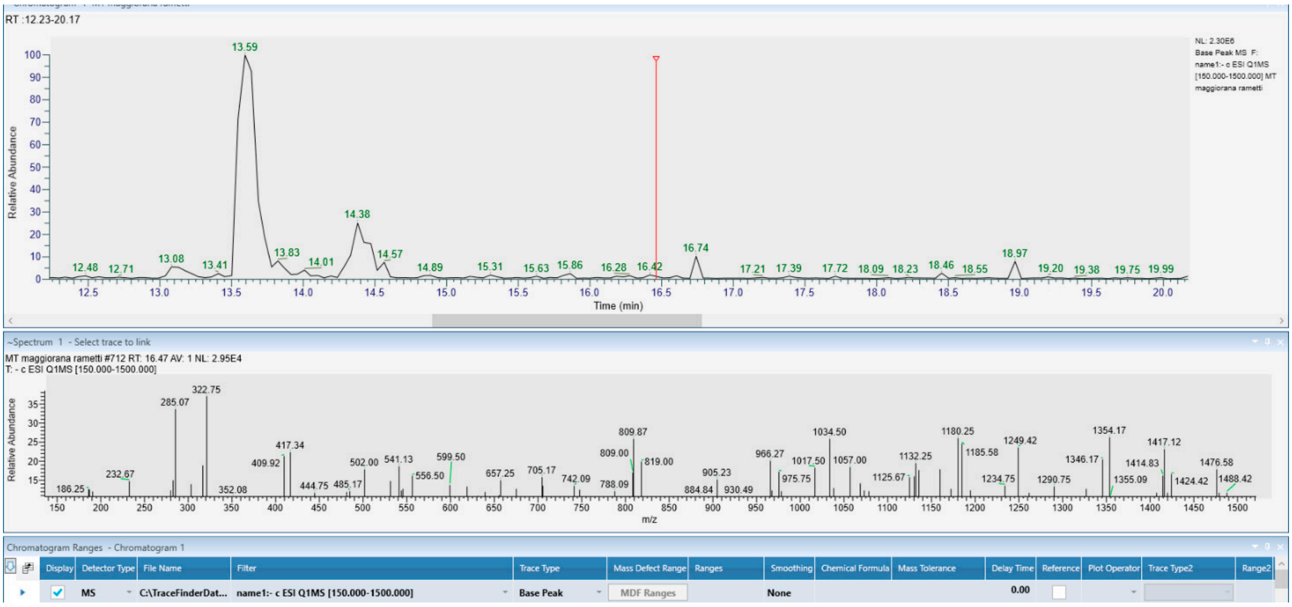

Peak 17, tentatively identified as di-p-coumaroyl acid isomer 2, MW = 484,1369; diagnostic ion $[M-H]^-$ and 190.92 m/z (quinate ion)

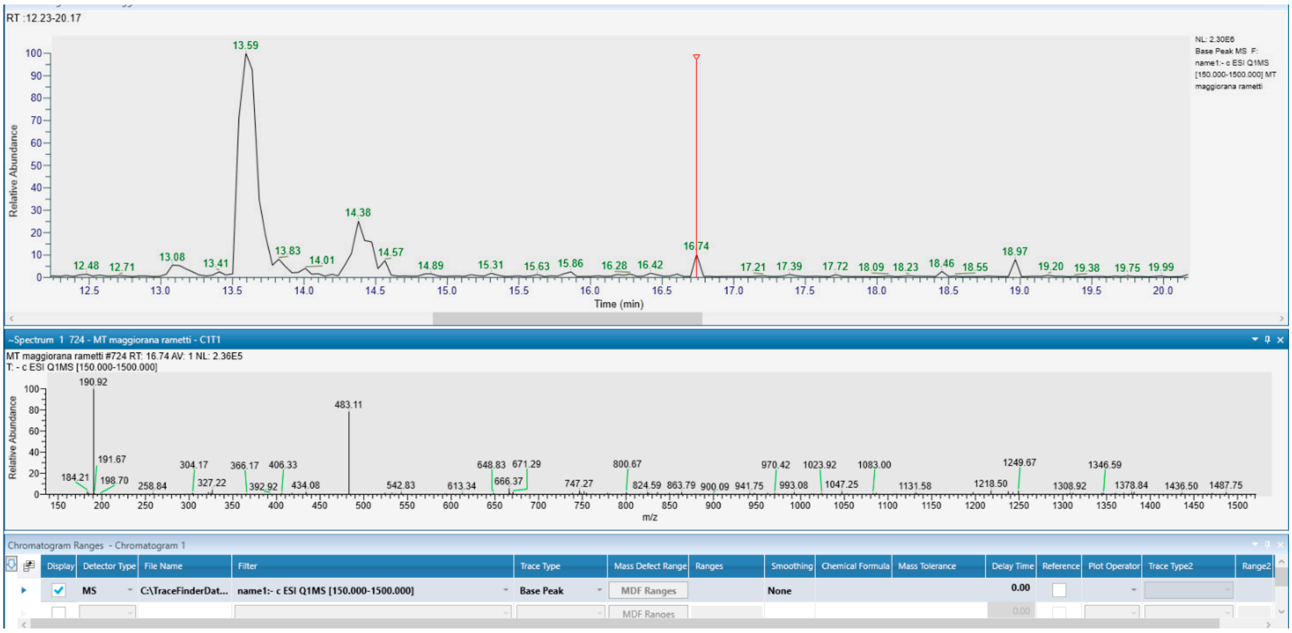

Peak 18, tentatively identified as salvianolic acid B isomer, MW = 718,1533; diagnostic ion $[M-H]^-$

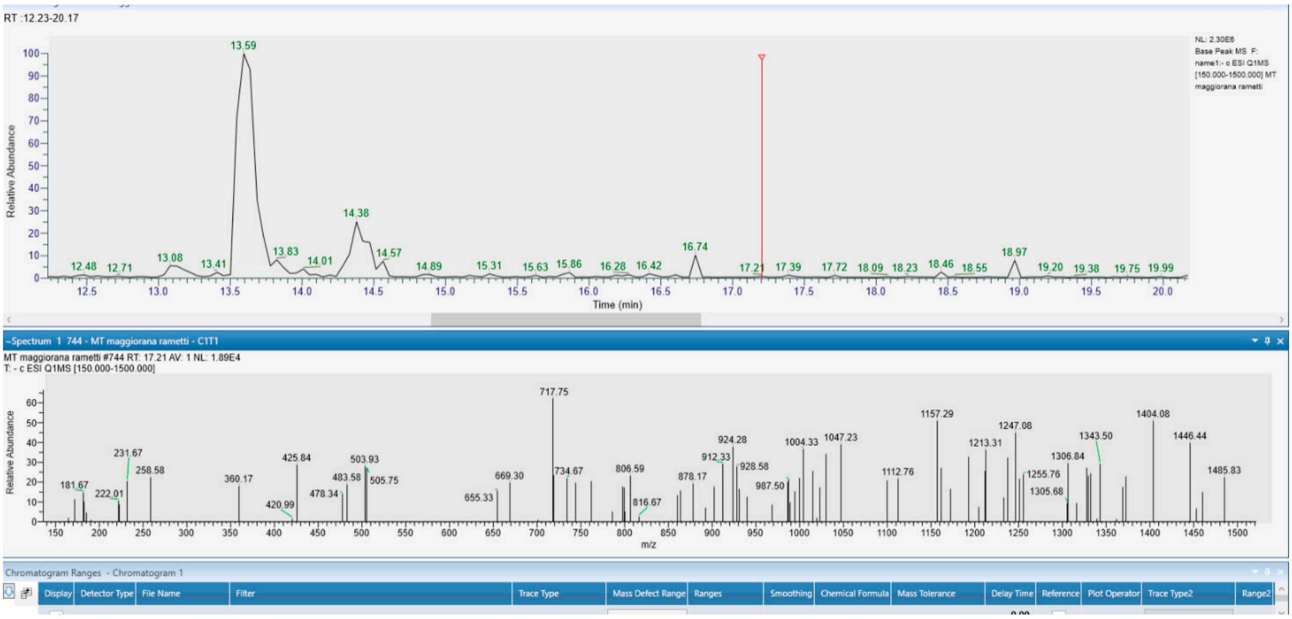

Peak 19, tentatively identified as dimethyl luteolin, MW = 314,079; diagnostic ion $[M-H]^-$

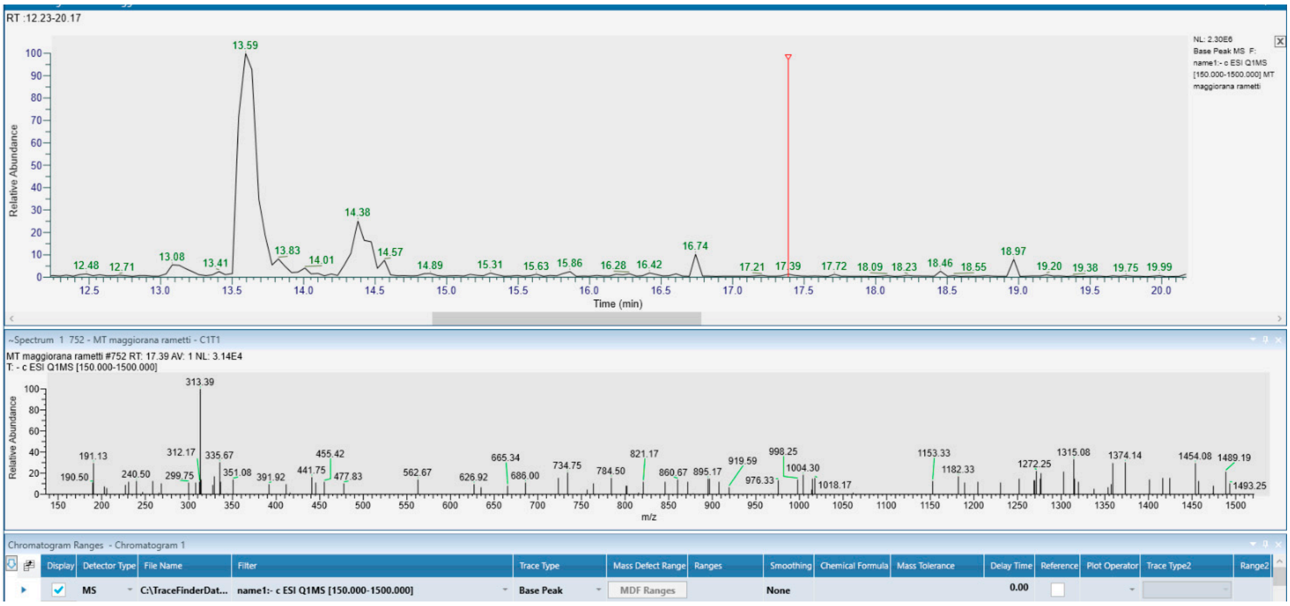

Peak 20, hesperetin, MW = 302,079; diagnostic ion $[M-H]^-$

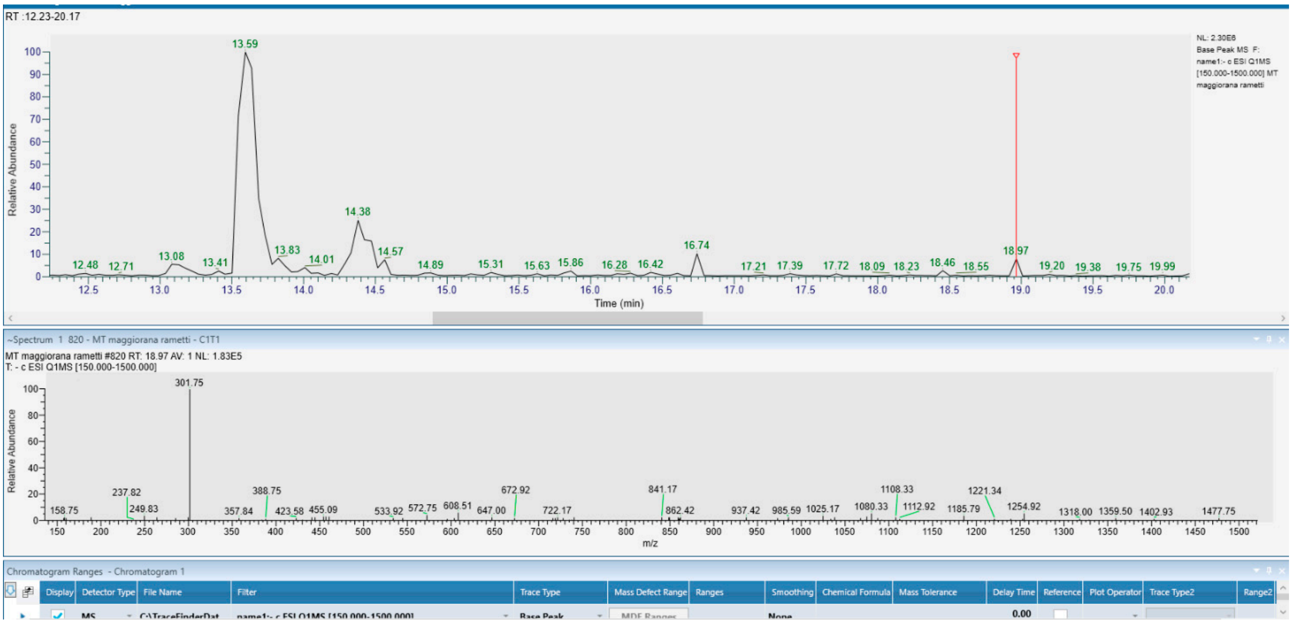

Supplement: Supplementary file 1 [file plants-14-02264-s001.zip › plants-3752048-supplementary.pdf]
